# The Double-Edged Sword of Genomic DNA Methylation: Orchestrating Gastric Carcinogenesis and Shaping Precision Oncology

**DOI:** 10.32604/or.2026.079753

**Published:** 2026-08-13

**Authors:** Xuan Chen, Chao Luo, Wei Wen

**Affiliations:** Department of Gastroenterology, Jiangsu Second Traditional Chinese Medicine Hospital, Nanjing, China

**Keywords:** DNA methylation, gastric cancer, epigenetics, precision oncology, biomarker, liquid biopsy, hypomethylating agents, immunotherapy

## Abstract

This article systematically elaborates on the dual role of DNA methylation in the initiation and progression of gastric cancer and its potential clinical applications in precision oncology. As a core epigenetic mechanism, DNA methylation drives the multistage development of gastric cancer through the coordinated dysregulation of genome-wide hypomethylation and promoter-specific hypermethylation, playing a key role in chronic inflammation and epigenetic reprogramming, particularly in the context of *Helicobacter pylori* infection. The article focuses on analyzing the central pathogenic mechanisms of DNA methylation, including the silencing of tumor suppressor genes, induction of genomic instability, promotion of the CpG island methylator phenotype, and facilitation of epithelial-mesenchymal transition. It further explores the clinical utility of DNA methylation as a biomarker for early diagnosis, prognosis evaluation, recurrence monitoring, and prediction of therapeutic response in gastric cancer. Moreover, the article reviews epigenetic therapeutic strategies represented by hypomethylating agents and their potential and challenges when combined with chemotherapy or immunotherapy, envisioning an integrated precision medicine model based on methylation profiling.

## 1 Introduction

Gastric cancer stands as a formidable global health challenge, ranking among the leading causes of cancer-related mortality worldwide [1]. Its high fatality rate is intrinsically linked to late-stage diagnosis and the limited efficacy of conventional therapies for advanced disease [2]. This clinical reality underscores an urgent need to deepen our molecular understanding of gastric carcinogenesis and to identify novel strategies for early intervention and targeted treatment [3]. For decades, the quest to unravel the biology of gastric cancer has largely navigated the terrain of genetic alterations, focusing on mutations, amplifications, and deletions within the DNA sequence itself. While this genetic landscape provides a crucial blueprint, it fails to fully explain the complex initiation and progression of the disease [4]. A significant piece of the puzzle lies in the dynamic and reversible layer of regulatory information superimposed upon the genetic code, a realm governed by epigenetics [5].

Among the various epigenetic mechanisms, DNA methylation reigns as a cornerstone, involving the covalent addition of a methyl group to the cytosine base within cytosine-phosphate-guanine dinucleotide sequences. This modification, meticulously orchestrated by DNA methyltransferases, does not alter the primary DNA sequence but exerts profound control over gene expression patterns [6]. In normal cellular physiology, DNA methylation plays pivotal roles in essential processes such as genomic imprinting, X-chromosome inactivation, and silencing of transposable elements, thereby maintaining genomic stability and cellular identity [7]. However, in the context of cancer, this precisely calibrated system becomes profoundly dysregulated, contributing directly to malignant transformation. The aberration manifests as a paradoxical and coordinated dual pattern [8]. On one edge of the sword, widespread genome-wide hypomethylation occurs, particularly at repetitive elements and intergenic regions [9]. This loss of methylation erodes genomic architecture, promotes chromosomal instability, and can inadvertently activate latent proto-oncogenes and endogenous retroviral elements. On the opposing edge, a contrasting process of focal, promoter-specific hypermethylation targets the CpG islands of numerous tumor suppressor genes. This hypermethylation acts as a molecular switch, hermetically silencing critical genes involved in cell cycle control, apoptosis, DNA repair, and cellular adhesion [10].

This double-edged dysregulation is not a random occurrence in gastric cancer but is often initiated and perpetuated by chronic environmental insults, most notably infection with *Helicobacter pylori* [11]. This bacterial pathogen acts as a potent epigenetic disruptor, inciting a state of chronic inflammation that remodels the methylome of gastric epithelial cells [12]. This process initiates a field defect, a precancerous landscape of epigenetic alterations that sets the stage for the classic Correa cascade, the stepwise progression from chronic gastritis through atrophy, intestinal metaplasia, dysplasia, and ultimately invasive adenocarcinoma [13]. At each histological stage, specific DNA methylation marks accumulate, serving as both drivers of malignant progression and potential biomarkers for risk stratification [14].

The discovery of distinct molecular subtypes of gastric cancer by comprehensive genomic profiling efforts, such as those by The Cancer Genome Atlas, has further cemented the central role of epigenetics. Notably, the Epstein-Barr virus positive subtype is characterized by an extreme degree of CpG island hypermethylation, designated the CpG Island Methylator Phenotype, which silences a vast array of genes [15]. Furthermore, methylation-mediated silencing of the *MLH1* mismatch repair gene is a key mechanism leading to microsatellite instability, another prominent molecular subtype with significant therapeutic implications [16]. These insights transition the role of DNA methylation from a peripheral phenomenon to a central pathogenic driver, intricately woven into the molecular fabric of gastric cancer subtypes [17].

Beyond its fundamental role in tumorigenesis, the very properties that make DNA methylation a potent driver of cancer also render it an exceptionally attractive target for clinical translation [18]. First, its chemical stability and the defined nature of CpG sites make methylation alterations highly detectable and quantifiable biomarkers [19]. Second, unlike irreversible genetic mutations, abnormal methylation patterns are, in principle, reversible, opening a therapeutic window for pharmacological intervention. Consequently, the clinical relevance of DNA methylation in gastric cancer is rapidly expanding across a triad of applications [20]. In diagnostics, methylation signatures in tissues and, more recently, in circulating cell-free DNA from blood liquid biopsies offer promise for non-invasive early detection and monitoring. In prognostication, specific methylation profiles correlate with clinical outcomes, guiding patient management [21]. Most excitingly, in therapeutics, drugs that inhibit DNA methyltransferases can reverse silencing of tumor suppressor genes and immunogenic viral elements, potentially restoring drug sensitivity and enhancing the efficacy of emerging immunotherapies [22].

**Figure 1:**
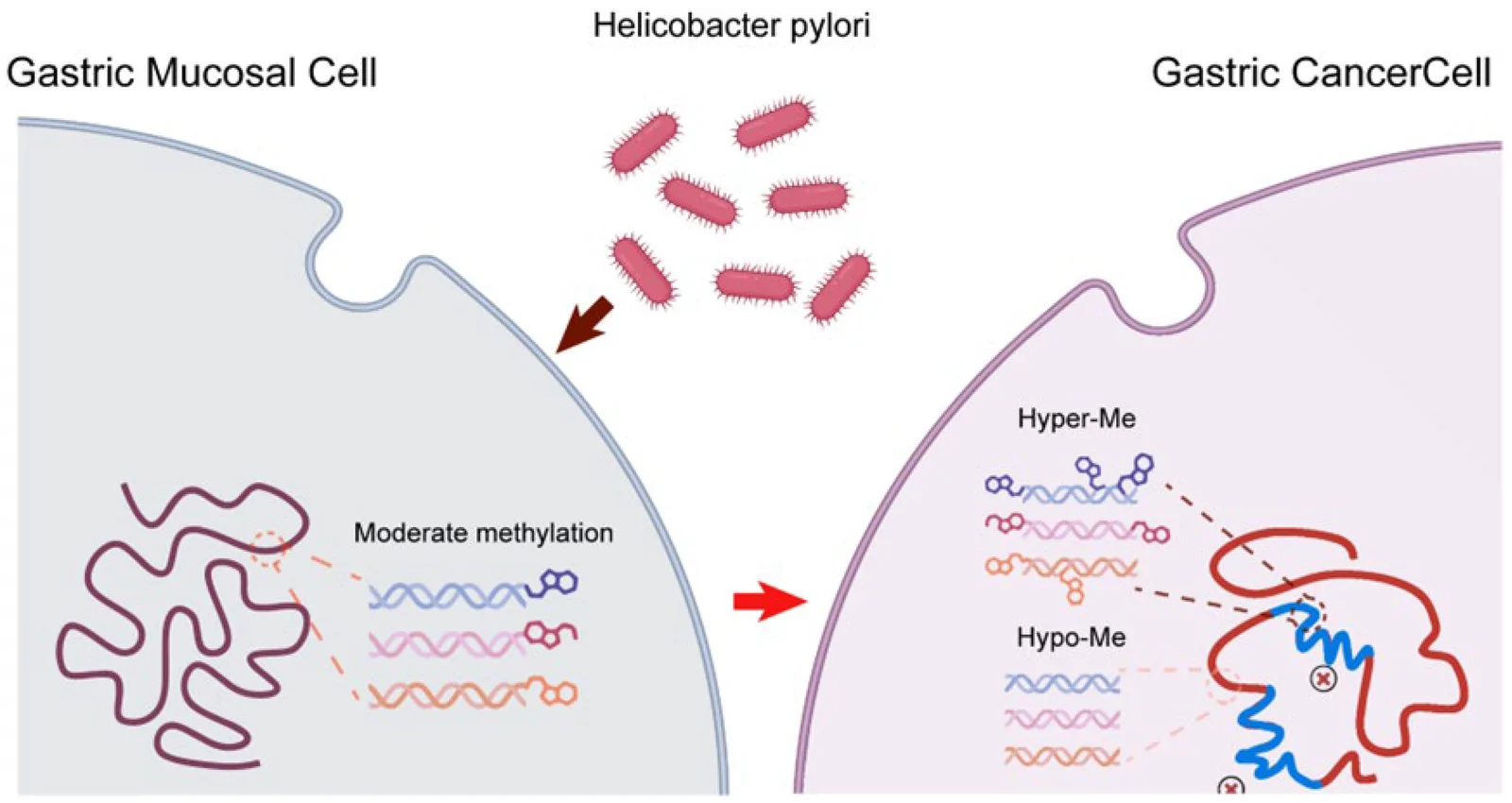
Comparison of DNA methylation in normal cells and gastric cancer cells. Figure Legend: Schematic representation of the proposed relationship among *H. pylori* infection, altered DNA methylation patterns in gastric mucosal cells, and progression to gastric cancer. Chronic infection with *H. pylori* is associated with dysregulated methylation, including both hypermethylation (Hyper-Me) and hypomethylation (Hypo-Me) events, which may contribute to the transformation of normal gastric mucosal cells into gastric cancer cells. Moderate methylation levels are observed in intermediate or transitional states. The model highlights the potential role of epigenetic modifications in *H. pylori*-induced gastric carcinogenesis. Original figure drawn independently using Adobe Illustrator (Version 29.7, Adobe Inc., USA, official website: https://www.adobe.com/).

Therefore, this review aims to comprehensively dissect the dual role of DNA methylation as a double-edged sword in gastric cancer [23]. We will delve into the mechanisms by which methylation aberrations orchestrate the multi-stage process of gastric carcinogenesis, from initial environmental insult to metastatic dissemination [24]. We will then critically examine how this knowledge is actively shaping the frontier of precision oncology, focusing on the development of methylation-based biomarkers for early detection and minimal residual disease monitoring, and on the strategic targeting of the methylome in combination with conventional and immune-based therapies [25]. By synthesizing insights from fundamental biology to clinical trials, this discussion seeks to illuminate the path toward integrating epigenetic strategies into the holistic management of gastric cancer, ultimately aiming to turn this molecular double-edged sword into a precise instrument for improving patient outcomes [26] (Fig. 1).

## 2 The Cascade of Aberrant DNA Methylation in Gastric Carcinogenesis

### 2.1 H. pylori Infection and Chronic Inflammation

The development of gastric cancer is not a sudden event but a multistep, progressive cascade. At the inception of this complex sequence, chronic infection and the ensuing inflammation play a pivotal role [27]. *H. pylori* infection, the strongest environmental risk factor, establishes a foundation for the epigenetic remodeling and malignant transformation of the gastric mucosa by inducing chronic active gastritis that can persist for decades [11]. This bacterium acts not merely as an inflammatory pathogen but as a potent epigenetic disruptor. Among its virulence factors, particularly in pathogenic strains, cytotoxin-associated gene A stands out as a core effector molecule responsible for inducing aberrant DNA methylation [4]. Upon injection into gastric epithelial cells via a type IV secretion system, cytotoxin-associated gene A activates a suite of critical intracellular signaling pathways, including nuclear factor kappa B and mitogen-activated protein kinase cascades [28]. This signal transduction leads to the sustained production of pro-inflammatory cytokines and directly or indirectly upregulates the activity and expression of DNA methyltransferases. The consequence is the *de novo* methylation of CpG islands within the promoter regions of specific genes [29]. The silencing of the mismatch repair gene *MLH1* serves as a classic example, directly leading to the emergence of the specific molecular phenotype known as microsatellite instability [30]. Furthermore, other genes involved in cell cycle control and apoptosis, such as *TYMS*, frequently become early targets of this bacterium-mediated methylation event [30]. Thus, through factors like cytotoxin-associated gene A, *H. pylori* functions as a trigger for epigenetic reprogramming, setting gastric mucosal cells on a path toward dysregulated gene expression [31].

A critical, yet often underappreciated, aspect of this process is the long-term persistence of certain methylation marks, even after the successful eradication of *H. pylori*. The biological basis for this persistence lies in the concept of epigenetic memory within long-lived gastric stem and progenitor cells. Unlike differentiated epithelial cells, which are continuously shed and replaced, gastric stem cells possess self-renewal capacity and extended lifespans, allowing acquired methylation patterns to be propagated through multiple cell divisions. These cells serve as permanent reservoirs of epigenetic abnormalities: once a methylation mark is established in a stem cell, it becomes heritable through DNA replication via the maintenance methyltransferase DNMT1 [32].

Importantly, the reversibility of methylation changes upon *H. pylori* eradication is gene-specific and context-dependent, demanding a clear distinction between reversible methylation changes and persistent field defects. Reversible changes typically occur in differentiated gastric epithelial cells. When the inflammatory trigger is removed, these short-lived cells are eventually replaced by newly differentiated, non-methylated progeny, explaining why methylation of certain genes (e.g., *CDH1*, p16, COX2) can decrease or disappear after successful eradication [29]. In contrast, persistent changes are those acquired in the gastric stem/progenitor cell compartment. These self-renewing cells enable the stable propagation of epigenetic abnormalities across generations, creating a permanent field defect. For instance, methylation of *MLH1* in patients with established intestinal metaplasia does not significantly change after eradication, suggesting that certain events become “fixed” in advanced precancerous lesions [33].

Thus, the persistence of *H. pylori*-associated methylation is not a deterministic guarantee of malignant progression but rather represents an increased risk state—an epigenetically primed field defect that creates a permissive microenvironment for subsequent genetic hits. This nuanced understanding is clinically important: it explains why eradication therapy significantly reduces but does not completely eliminate gastric cancer risk, and it underscores the need for risk-stratified surveillance strategies targeting individuals with evidence of persistent field defects [34].

This concept of a persistent, epigenetically altered field directly underpins field cancerization, wherein histologically normal tissue harbors accumulated molecular abnormalities that confer elevated cancer risk [35]. Field cancerization describes a histologically appearing normal tissue field that has already accumulated molecular abnormalities, granting it a higher risk of malignant transformation. DNA methylation markers serve as ideal indicators of such molecular defects [3]. Research shows that abnormal methylation patterns similar to those in gastric cancer can be detected in gastric mucosa that has not yet undergone histological cancerous change, particularly in individuals infected with *H. pylori*. These methylation changes, such as silencing marks on tumor suppressor genes, persist as molecular scars. Even after successful eradication of *H. pylori*, some methylation patterns may endure [36]. This persistence explains why eradication therapy significantly reduces but does not completely eliminate gastric cancer risk in previously infected individuals. These pre-existing, scattered epigenetic abnormalities across a susceptible field constitute a fertile soil for cancer development [37]. In this sense, DNA methylation is not only a driver of cancer but also an early molecular gauge that records and defines the precancerous field effect [38]. It provides a crucial clue for understanding how cancer gradually evolves from a field of epigenetically damaged tissue and suggests that interventions targeting these early methylation events may hold preventive value [39]. Therefore, the inflammation triggered by *H. pylori* and the accompanying methylation reprogramming together form the irreversible opening movement in the carcinogenic cascade of gastric cancer [40] (Fig. 1).

### 2.2 Evolution of the Methylation Landscape along the Correa Cascade

Following the initial insult by *H. pylori*, the gastric mucosa embarks on a well-defined pathological journey known as the Correa cascade, a stepwise progression from chronic gastritis to invasive adenocarcinoma. This histological transformation is paralleled and likely driven by a dynamic and cumulative reprogramming of the DNA methylome. The methylation landscape does not shift abruptly but evolves in a gradual manner, with specific alterations marking and potentially propelling each precancerous stage [41].

The first significant step is chronic atrophic gastritis, characterized by the loss of specialized gastric gland cells. This stage witnesses the emergence of a more pronounced and organized aberrant methylation pattern. While the initial *H. pylori*-induced changes might be somewhat scattered, atrophy is associated with the targeted silencing of genes crucial for normal gastric differentiation and function [42]. For instance, genes involved in acid secretion and mucosal defense begin to show increased promoter methylation [43]. This epigenetic silencing contributes to the functional loss of the gastric epithelium, creating a permissive microenvironment. Importantly, the degree of methylation at this stage, particularly in specific gene panels, has been proposed as a quantitative biomarker to assess the extent of atrophy and stratify cancer risk, offering a molecular mirror to the histological changes [44].

The process advances with intestinal metaplasia, where the gastric lining is replaced by intestinal-type epithelium. This morphological shift is underpinned by a profound epigenetic switch. Methylation changes during metaplasia often target promoters of genes that are key to maintaining gastric cell identity, effectively silencing the gastric genetic program [45]. Concurrently, there is evidence of hypomethylation at loci associated with intestinal differentiation, facilitating this cellular reprogramming [45]. The methylation profile of metaplastic tissue becomes more complex and starts to overlap significantly with that of frank carcinomas [46]. Certain methylation marks, such as the silencing of specific developmental transcription factors, are so strongly associated with metaplasia that they are considered hallmarks of this stage, solidifying its role as a critical point of no return in the carcinogenic pathway [46].

The transition to dysplasia, or intraepithelial neoplasia, represents the penultimate stage before invasion and is marked by overt cellular and architectural atypia. The methylation landscape at this stage intensifies and converges toward a clearly neoplastic signature [47]. There is a marked increase in the density and number of hypermethylated genes, particularly those involved in robust tumor suppression, cell adhesion, and DNA damage response. The global hypomethylation that promotes genomic instability also becomes more pronounced. This combination creates a powerful epigenetic drive for uncontrolled proliferation and impaired DNA repair. In high-grade dysplasia, the methylation patterns are virtually indistinguishable from invasive cancer, underscoring its status as a direct precursor [48]. The epigenetic alterations in dysplasia are not merely passenger events but are functionally implicated in conferring the hallmark capabilities of pre-malignant cells [49].

Finally, the culmination of this multistep process is invasive adenocarcinoma. The methylation profile of the established carcinoma is a complex mosaic, reflecting its clonal evolution and heterogeneity [50]. It carries forward and amplifies all prior alterations: widespread promoter hypermethylation of tumor suppressor genes, extensive global hypomethylation leading to genomic chaos, and the characteristic CpG Island Methylator Phenotype observed in specific molecular subtypes like EBV-positive tumors. New layers of methylation dysregulation may also emerge, driving processes such as epithelial-mesenchymal transition and metastasis through the silencing of cell adhesion molecules like E-cadherin. At this stage, the methylome is both a historical record of the carcinogenic journey and an active engine of tumor progression, maintaining the malignant phenotype and contributing to therapeutic resistance.

Importantly, while the Correa cascade provides an invaluable histopathological framework, recent evidence cautions against viewing it as a universal or strictly linear sequence. First, an alternative metaplastic lineage—spasmolytic polypeptide-expressing metaplasia (SPEM)—has been identified as a parallel pathway that may precede or coexist with intestinal metaplasia. SPEM arises from chief cell transdifferentiation or stem cell reprogramming following parietal cell loss and, under chronic inflammatory conditions, can progress to dysplasia independently of the classic intestinal metaplasia route [51]. Second, genomic profiling studies have revealed that molecular alterations do not always accumulate in a stepwise fashion. For instance, TP53 mutations can already be detected at the low-grade intraepithelial neoplasia stage, and intestinal metaplasia shows no significant mutational correlation with early gastric cancer in some cohorts, suggesting that the “point of no return” may occur earlier than traditionally appreciated and that different precursor lesions may give rise to cancer through convergent evolution [52]. Third, mixed-type gastric cancer (exhibiting both intestinal and diffuse features) demonstrates intratumoral heterogeneity that cannot be explained by a single linear trajectory [53]. These nuances do not invalidate the Correa cascade but rather enrich our understanding of gastric carcinogenesis as a complex, multi-pathway process [51]. They also have direct clinical implications: biomarker panels must account for pathway heterogeneity, early detection strategies might target SPEM as an earlier intervention point, and precision oncology approaches need to accommodate both convergent and divergent evolutionary dynamics [54].

**Figure 2:**
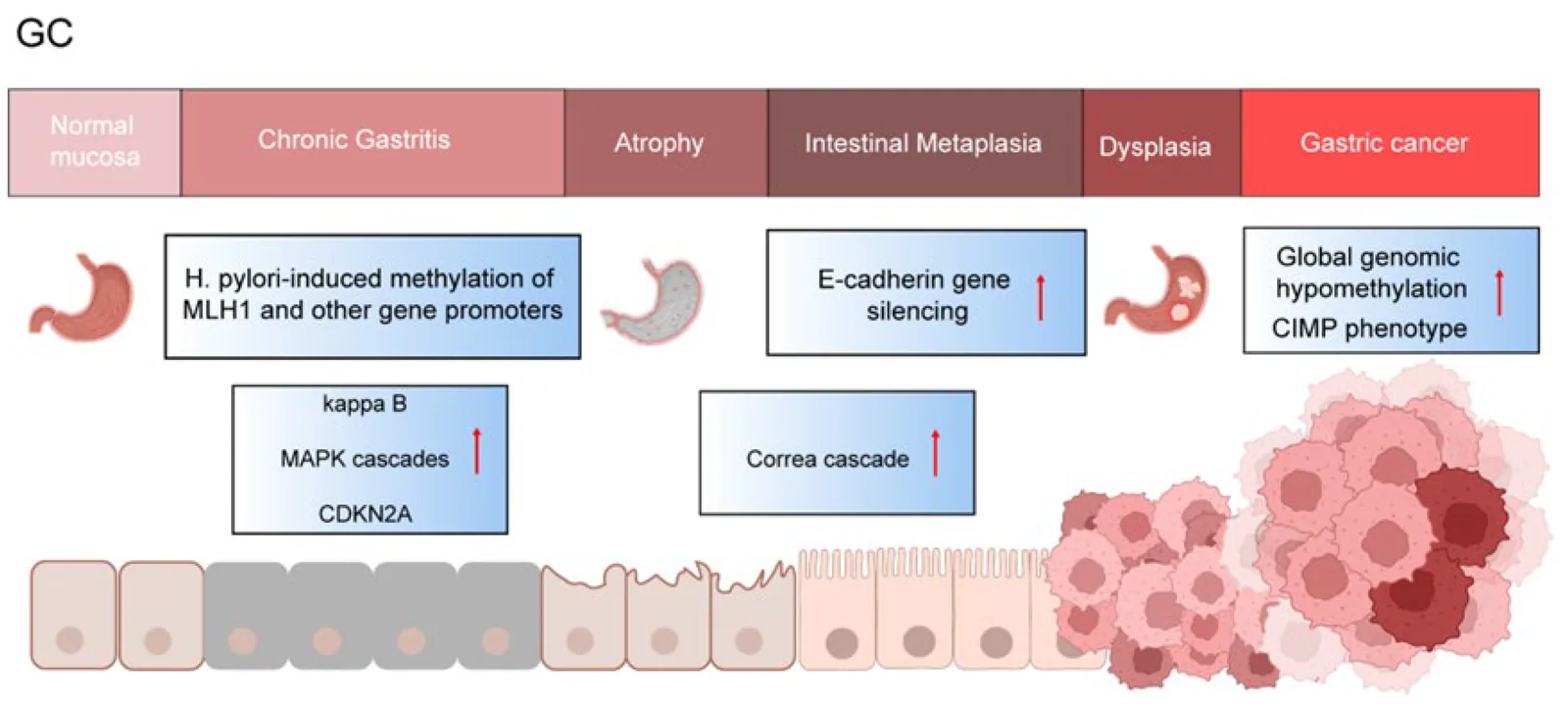
Cascade reaction and key mechanism of abnormal DNA methylation driving gastric cancer occurrence. Timeline depicting the stepwise pathological progression of gastric carcinogenesis (Correa cascade) from normal mucosa to gastric cancer (GC). Arrow 1 (between Normal mucosa and Chronic Gastritis): Indicates the initiation of the Correa cascade, where H. pylori infection triggers the first wave of epigenetic alterations (e.g., MLH1 promoter methylation) even before overt histological changes. Arrow 2 (between Chronic Gastritis and Atrophy): Represents the progression of both pathological severity and methylation burden, marking the transition to chronic atrophic gastritis. Arrow 3 (between Atrophy and Intestinal Metaplasia): Denotes the critical transition point, where epigenetic silencing (e.g., E-cadherin) drives cellular reprogramming toward an intestinal phenotype. Arrow 4 (between Intestinal Metaplasia and Dysplasia): Signifies the acceleration of epigenetic dysregulation, characterized by increasing global hypomethylation and CIMP emergence, propelling the mucosa toward frank neoplasia. The four upward arrows between successive stages collectively indicate the progressive, unidirectional, and cumulative nature of the Correa cascade. Beneath each histopathological stage, key epigenetic alterations driven by *H. pylori* infection are shown. These methylation events accumulate progressively: *H. pylori*-induced promoter methylation of genes, including *MLH1*, occurs during chronic gastritis; E-cadherin gene silencing arises at the intestinal metaplasia stage. In advanced stages, global genomic hypomethylation and the CpG island methylator phenotype (CIMP) become prominent, accompanied by activation of inflammatory pathways such as NF-κB and MAPK cascades, and inactivation of tumor suppressor genes, including *TYMS*. The figure illustrates the integration of histopathological progression with underlying epigenetic and molecular mechanisms in gastric cancer development. Original figure drawn independently using Adobe Illustrator (Version 29.7, Adobe Inc., USA, official website: https://www.adobe.com/).

The sequential and additive nature of methylation changes across the Correa cascade provides compelling evidence that epigenetic dysregulation is a fundamental driver, not a late consequence, of gastric neoplasia [55] (Fig. 2).

### 2.3 Epstein-Barr Virus Infection as a Potent Epigenetic Driver

While *H. pylori* represents the most prevalent bacterial risk factor for gastric cancer, viral pathogens also play a significant etiological role, with Epstein-Barr virus (EBV) being the most well-characterized. EBV-associated gastric cancer (EBVaGC) accounts for approximately 8–10% of all gastric cancer cases globally and exhibits a distinct molecular landscape defined by extreme epigenetic dysregulation [15].

Unlike *H. pylori*, which induces a gradual accumulation of methylation changes over decades, EBV infection drives a more profound and rapid epigenetic reprogramming. The virus establishes latent infection in gastric epithelial cells, during which viral latency proteins (such as LMP2A and EBNA1) and non-coding RNAs (including EBERs and BART miRNAs) interact with the host epigenetic machinery. These viral factors recruit DNA methyltransferases (DNMTs) to specific genomic loci, leading to widespread and concerted CpG island hypermethylation [56].

This process results in the CpG Island Methylator Phenotype (CIMP), which is most extreme in EBVaGC. The hypermethylation landscape in these tumors silences a vast array of tumor suppressor genes, including *TYMS* (p16), PTEN, and the mismatch repair gene *MLH1*, contributing to genomic instability and uncontrolled proliferation. Notably, EBV-mediated methylation also frequently targets genes involved in immune signaling, paradoxically contributing to immune evasion while simultaneously rendering these tumors exquisitely sensitive to immune checkpoint inhibitors. The high PD-L1 expression characteristic of EBVaGC, partly driven by epigenetic mechanisms, further supports the clinical observation that these patients derive substantial benefit from immunotherapy.

Thus, EBV infection represents a paradigm of pathogen-driven epigenetic oncogenesis, distinct from but complementary to the *H. pylori*-induced field defect. The recognition of EBVaGC as a distinct molecular subtype with a unique methylation signature has profound implications for diagnosis, prognosis, and the selection of targeted immunotherapeutic strategies.

## 3 Key Pathogenic Mechanisms

The aberrant DNA methylation patterns observed across the gastric carcinogenesis cascade are not mere epiphenomena but act through distinct, powerful mechanisms to confer hallmark cancer capabilities [57]. These mechanisms operate in concert, dismantling cellular defense systems and activating pro-tumorigenic processes, thereby solidifying the role of the methylome as a central oncogenic driver.

### 3.1 Tumor Suppressor Gene Silencing

A principal oncogenic consequence of promoter-specific hypermethylation is the heritable transcriptional silencing of tumor suppressor genes [58]. However, it is critical to recognize that this relationship is neither binary nor absolute. In gastric cancer, as in other malignancies, promoter methylation patterns are frequently incomplete and heterogeneous, with individual CpG sites within a given promoter showing variable methylation levels across different tumor cells and even within the same tumor due to subclonal evolution [59,60]. Complete methylation of all CpG residues in a promoter region is not required for functional effects; rather, methylation at critical regulatory sites can be sufficient to impair transcription factor binding [61]. Moreover, the functional consequences of promoter methylation are profoundly contingent on the pre-existing chromatin context. A particularly instructive finding comes from studies of Polycomb group protein (PcG)-marked promoters. Promoters marked by PcG proteins in normal tissues are preferentially targeted for *de novo* hypermethylation in cancer, yet—paradoxically—frequently remain transcriptionally active [62,63]. This “PcG+ paradox” indicates that methylation’s repressive effect is not absolute but contingent on the underlying chromatin landscape. Furthermore, DNA methylation does not operate in isolation; transcriptional silencing often requires the concerted action of promoter hypermethylation with repressive histone modifications (e.g., H3K27me3) and the absence of active marks (e.g., H3K4me3) [64,65]. With these complexities acknowledged, several genes stand as well-validated examples of methylation-associated silencing in gastric cancer. *RASSF1A*, a key regulator of microtubule stability and apoptotic signaling, is frequently methylated in gastric tumors [11]. Its silencing disrupts mitotic progression and impairs the cellular response to pro-apoptotic signals, promoting unchecked proliferation. Similarly, the runt-related transcription factor 3, *RUNX3*, a crucial regulator of transforming growth factor beta-induced growth inhibition and apoptosis, is commonly inactivated via methylation [4]. Loss of *RUNX3* function renders cells insensitive to growth-suppressive cues. The *CDH1* gene, encoding the cell adhesion protein E-cadherin, represents another critical target. Its methylation-mediated silencing is a pivotal event in the loss of epithelial cohesion, directly facilitating cellular detachment, invasion, and the initiation of metastasis. Finally, methylation of the *MLH1* promoter is a cornerstone event in a subset of gastric cancers [66]. *MLH1* is essential for DNA mismatch repair, and its silencing leads to high microsatellite instability, a phenotype characterized by widespread mutations and which carries significant implications for prognosis and therapy response [67]. The collective silencing of these and numerous other gatekeeper genes across diverse pathways creates a permissive cellular environment for tumorigenesis (Table 1).

**Table 1:** Key tumor suppressor genes silenced by promoter hypermethylation in gastric cancer.

Gene	Functional Pathway	Methylation Frequency in GC	Clinical Association	Reference
*RASSF1A*	Apoptosis, Microtubule Stability, Cell Cycle	High (40–60%)	Associated with poor differentiation, advanced stage; an independent prognostic factor for poorer survival.	[68]
*RUNX3*	TGF-β Signaling, Apoptosis, Growth Inhibition	High (30–70%)	Correlates with tumor progression, lymph node metastasis, and serves as a prognostic marker.	[69]
*CDH1* (E-cadherin)	Cell-Cell Adhesion, Invasion Suppression	Variable (20–50%, higher in diffuse-type)	Hallmark of diffuse-type GC; strongly linked to EMT, invasion, and hereditary diffuse GC.	[70]
*MLH1*	DNA Mismatch Repair	~15–20% of sporadic GC	Causative for MSI-H phenotype; biomarker for immunotherapy response and favorable prognosis in early stages.	[71]
*TYMS* (p16)	Cell Cycle Control (G1/S checkpoint)	High (30–50%)	Associated with Lauren intestinal type; early event in carcinogenesis; prognostic marker.	[72]
SFRP1/2	WNT Signaling Pathway (Extracellular Inhibitor)	High (SFRP1: ~50%; SFRP2: ~40%)	Frequent early event; leads to constitutive WNT activation; associated with poor prognosis.	[73]
DAPK	Apoptosis, Autophagy	Moderate to High (30–60%)	Associated with advanced stage, metastasis, and poorer survival; a potential therapeutic target.	[74]
*MGMT*	DNA Repair (Alkylation Damage)	Moderate (20–40%)	Predicts sensitivity to alkylating agents (e.g., temozolomide); associated with specific mutation signatures.	[75]
CHFR	Mitotic Checkpoint, Cell Cycle	Moderate (~30–50%)	Associated with chromosomal instability; potential predictor of taxane sensitivity.	[76]
RASSF2	RAS Signaling Inhibitor, Apoptosis	Moderate (30–50%)	Member of the RASSF family; correlates with tumor size and advanced stage; cooperative role with *RASSF1A*.	[77]

**Note:** EMT, epithelial-mesenchymal transition; GC, gastric cancer; MSI-H, microsatellite instability-high; dMMR, mismatch repair-deficient; TGF-β, transforming growth factor-beta.

DNA methylation plays a direct and instrumental role in facilitating cancer spread by driving the epithelial-mesenchymal transition (EMT). EMT is a developmental program co-opted by carcinoma cells to lose epithelial features like adhesion and polarity and gain migratory, invasive mesenchymal traits. Hypermethylation serves as a stable switch to silence critical epithelial maintainers. The silencing of the *CDH1* gene, which encodes E-cadherin, is the canonical example, leading to the dissolution of adherens junctions. Beyond E-cadherin, hypermethylation targets other epithelial integrity genes, such as those encoding cytokeratins and components of tight junctions. This coordinated epigenetic repression enables cells to detach from the primary tumor, invade the surrounding stroma, and ultimately metastasize. The stability of methylation marks ensures that these pro-metastatic phenotypic changes are heritably maintained in disseminating tumor cells, contributing to the establishment of lethal secondary tumors.

#### Context-Dependent Consequences of Promoter Methylation

The functional impact of promoter methylation depends critically on several layers of context, moving beyond a simple binary model.

CpG island versus non-CpG island promoters. Promoters can be stratified by CpG density. CpG island promoters, typically unmethylated in normal cells, show increased methylation in cancer—a feature associated with transcriptional repression of tumor suppressors. In contrast, non-CpG island promoters, normally methylated in healthy tissues, often become hypomethylated in tumors, potentially activating oncogenes [78,79]. The functional consequences thus differ fundamentally based on promoter type.

Polycomb group protein occupancy as a modifier. As discussed above, PcG+ promoters exhibit a paradoxical resistance to methylation-induced silencing [62]. Single-cell analyses have revealed that within a single tumor, subpopulations of cells can harbor distinct methylation patterns at the same locus, leading to heterogeneous gene expression that may facilitate adaptation and therapeutic resistance [59].

Cooperative epigenetic mechanisms. DNA methylation does not operate in isolation. The combination of promoter hypermethylation with repressive histone modifications (e.g., H3K27me3) creates a more stable silencing state than either modification alone [64]. In gastric cancer, studies have demonstrated that transcriptional silencing can require both epigenetic layers [65].

These contextual dependencies have profound implications for interpreting methylation data. Methylation of a promoter does not guarantee gene silencing; the functional outcome depends on methylation density, location of methylated CpGs relative to transcription factor binding sites, and chromatin state [80]. Future studies must move beyond binary methylation calls toward quantitative, context-aware assessments.

### 3.2 Genomic Instability

Opposing the focal hypermethylation is a genome-wide loss of methylation, particularly at repetitive DNA elements such as Long Interspersed Nuclear Element 1 sequences. This global hypomethylation erodes genomic architecture. In normal cells, methylation of these repetitive sequences suppresses their recombination and transposition [81]. Their demethylation in gastric cancer leads to chromosomal instability, including translocations, deletions, and amplifications. Furthermore, hypomethylation can directly activate silenced proto-oncogenes or facilitate their overexpression by altering chromatin structure at their regulatory regions. This combination of increased mutational burden from structural rearrangements and the aberrant activation of growth-promoting genes creates a potent engine for genetic diversity and clonal evolution, fueling tumor heterogeneity and adaptation [82].

### 3.3 CpG Island Methylator Phenotype

A subset of gastric cancers exhibits a concerted hypermethylation of numerous CpG islands, a feature termed the CpG Island Methylator Phenotype. CIMP is not a random event but is tightly linked to specific molecular etiologies [83]. It is most prominently and intensely observed in Epstein-Barr virus-positive gastric cancers, where viral factors are thought to drive widespread epigenetic silencing. CIMP is also a hallmark of microsatellite unstable tumors, often initiated by the methylation-mediated silencing of the *MLH1* gene [84]. This phenotype represents an extreme form of epigenetic dysregulation, where the simultaneous silencing of hundreds of genes, many involved in development and differentiation, defines a distinct biological and clinical subgroup with unique vulnerabilities.

### 3.4 Epithelial-Mesenchymal Transition and Metastasis

DNA methylation plays a direct and instrumental role in facilitating cancer spread by driving the epithelial-mesenchymal transition. EMT is a developmental program co-opted by carcinoma cells to lose epithelial features like adhesion and polarity and gain migratory, invasive mesenchymal traits. Methylation serves as a stable switch to silence critical epithelial maintainers. The silencing of the *CDH1* gene, as mentioned, is the canonical example, leading to the dissolution of adherens junctions. Beyond E-cadherin, methylation targets other epithelial integrity genes, such as those encoding cytokeratins and components of tight junctions. This coordinated epigenetic repression enables cells to detach from the primary tumor, invade the surrounding stroma, and ultimately metastasize [85]. The stability of methylation marks ensures that these pro-metastatic phenotypic changes are heritably maintained in disseminating tumor cells, contributing to the establishment of lethal secondary tumors [81].

### 3.5 Mechanistic Relationship between Hypermethylation and Hypomethylation

A critical conceptual question arising from the dual dysregulation of the methylome is whether promoter-specific hypermethylation and global hypomethylation represent mechanistically linked events or parallel, independent phenomena. Is the relationship between hypermethylation and hypomethylation a linear progression or an alternative evolution model?This distinction has important implications for understanding the fundamental biology of gastric carcinogenesis and for developing rational therapeutic strategies.

#### 3.5.1 Linked or Parallel Phenomena?

Accumulating evidence suggests that these two processes may arise through partially distinct molecular mechanisms. Promoter CpG island hypermethylation is primarily driven by the aberrant recruitment of DNA methyltransferases (DNMTs) to specific genomic loci. Many hypermethylated genes in gastric cancer are normally marked by polycomb repressive complex (PRC) components in embryonic stem cells, and this “bivalent” chromatin state may predispose them to aberrant methylation in cancer [83]. In contrast, global hypomethylation, particularly at repetitive elements such as LINE-1 sequences, is associated with inadequate maintenance of methylation during DNA replication, potentially due to age-related declines in DNMT1 fidelity or replication stress [81].

Despite their independence, emerging evidence suggests potential mechanistic links between these two phenomena. First, the genomic instability driven by global hypomethylation may indirectly promote hypermethylation by disrupting the expression or function of epigenetic modifiers. Second, both processes may converge downstream of common upstream drivers. Chronic inflammation induced by *H. pylori* infection, for instance, can simultaneously upregulate DNMT expression (promoting hypermethylation) and induce oxidative stress that impairs maintenance methylation (contributing to hypomethylation) [31]. Third, the two processes may be temporally coordinated: hypermethylation of specific tumor suppressor genes often occurs early in carcinogenesis, establishing a “field defect,” while global hypomethylation tends to accumulate progressively with advancing disease stage.

#### 3.5.2 Linear vs. Alternative Evolutionary Models?

The aberrant DNA methylation patterns observed across the gastric carcinogenesis cascade are not mere epiphenomena but act through distinct, powerful mechanisms to confer hallmark cancer capabilities. Importantly, these mechanisms do not operate in a simple linear sequence—with promoter hypermethylation preceding global hypomethylation—but rather reflect a coordinated, often simultaneous, reprogramming of the methylome. Recent comprehensive methylome analyses have revealed that CpG island hypermethylation and CpG-poor region hypomethylation are not independent events but represent a coordinated epigenetic landscape that emerges simultaneously from the early stages of neoplastic transformation. In normal-appearing gastric mucosa of patients who subsequently develop post-eradication gastric cancer, substantial CpG island hypermethylation is already detectable, accompanied by hypomethylation at approximately 4000 CpG sites—many of which are located near oncogenes such as *CDH17*, HNF4A, and CD44, with methylation levels inversely correlated with gene expression. Critically, a strong positive correlation exists between these two classes of alterations, suggesting that they are driven by common upstream mechanisms rather than independent sequential processes [5].

Furthermore, mechanistic studies have identified that a single molecular alteration—deletion of the replication foci targeting sequence (RFTS) domain of DNMT1—can reprogram both focal hypermethylation and global hypomethylation in parallel during malignant transformation [86]. This finding provides compelling evidence that these seemingly opposing epigenetic changes may share a common mechanistic origin rather than representing distinct temporal stages.

#### 3.5.3 Focal Hypermethylation and Cloning of Heterogeneous Precursor Cell Populations and Epigenetic Diverse Cell States

However, the occurrence of gastric cancer may result from heterogeneous precursor cell populations and epigenetic diverse cell states rather than from a single uniform methylation trajectory. Under chronic inflammatory conditions, partially dedifferentiated or replication-stressed epithelial cells are already unstable or have overall methylation maintenance changes, and may also serve as permissible substrates for subsequent focal hypermethylation and clonal selection.

Recent advances in organoid technology have provided direct experimental evidence for this model. Studies using human gastric intestinal metaplasia organoids have revealed that metaplastic epithelium spans a spectrum from hybrid gastric/intestinal differentiation to advanced intestinal differentiation, with single-cell transcriptomes demonstrating multiple coexisting lineage trajectories [86]. Critically, these organoid studies identified a cell-matrix adhesion-independent subpopulation that displays chromosome 20 gain but lacks key cancer driver mutations, potentially representing the earliest neoplastic precursor [87].

This discovery confirms that some dedifferentiated or replication-stressed epithelial cells may serve as permissive substrates for subsequent epigenetic alterations. It is worth noting that these precursor populations had already exhibited epigenetic dysregulation of multiple intestinal and gastric genes before obtaining classical driver mutations, suggesting that the epigenetic “pre-start” state may precede and promote clonal expansion.

The cellular origin of these coordinated alterations is equally complex. Human gastric intestinal metaplasia organoids have revealed that precancerous epithelium is not a homogeneous entity but spans a spectrum from hybrid gastric/intestinal differentiation to advanced intestinal differentiation, with multiple lineage trajectories coexisting within the same tissue [86]. Single-cell transcriptomics has identified distinct cycling and quiescent stem and progenitor populations, including STMN1+ cycling isthmus stem cells that may serve as the cell of origin for intestinal metaplasia [86]. Among these populations, hybrid metaplastic cells exhibit impaired differentiation potential, high lineage plasticity beyond gastric or intestinal fates, and reactivation of a fetal gene program—features that render them particularly susceptible to epigenetic deregulation [86]. Importantly, a subset of metaplastic organoids develops a cell-matrix adhesion-independent subpopulation that acquires chromosome 20 gain in the absence of canonical cancer driver mutations, representing the earliest neoplastic precursor [87]. These epigenetically primed cells exhibit deregulated methylation of multiple intestinal and gastric genes prior to malignant transformation.

The vulnerability of these precursor populations to epigenetic disruption is exacerbated by chronic inflammation. Oxidative stress, a hallmark of *H. pylori*-induced gastritis, directly impairs methylation maintenance through multiple mechanisms. Chloramines produced by activated neutrophils inhibit DNMT1 activity and deplete S-adenosylmethionine, the universal methyl donor, leading to site-specific decreases in DNA methylation that preferentially affect replicating cells and chromosomal end regions [54]. This cell cycle dependence explains why replication-stressed epithelial cells within inflamed mucosa are particularly susceptible to methylation instability. The resulting epigenetic heterogeneity creates a diverse landscape upon which selective pressures act. Experimental evolution studies using TP53-deficient gastric organoids have demonstrated that initially rare subclones with shared transcriptional programs repeatedly attain clonal dominance, revealing stringent selection and clonal interference during premalignant progression [88]. This process leads to marked phenotypic convergence despite diverse epigenetic starting states, explaining the emergence of stereotyped methylation patterns—such as the CpG island methylator phenotype—in established gastric cancers [5].

Thus, the relationship between hypermethylation and hypomethylation is usually coordinated and complex, and cannot be fully captured through a single linear timeline or parallel relationship. On the contrary, these changes arise from the dynamic interactions among common mechanism drivers (such as DNMT1 dysregulation, oxidative stress), heterogeneous cellular substrates (such as mixed stem cells, replication stress progenitor cells), and clonal selection pressure that shapes the epigenetic landscape in evolution. This integrated model accommodates both synergy and heterogeneity, providing a more accurate framework for understanding gastric cancer and developing epigenetic biomarkers and therapies.

## 4 DNA Methylation as a Clinical Biomarker in Gastric Cancer

### 4.1 Diagnosis and Early Detection

The high mortality rate of gastric cancer is predominantly linked to late-stage diagnosis, a point at which therapeutic options become severely limited and prognosis is often unfavorable [89]. Consequently, the development of robust biomarkers that enable accurate and early detection stands as a critical objective for improving patient outcomes. DNA methylation has emerged as a particularly promising candidate for this role, owing to its early occurrence in the carcinogenic cascade, its inherent chemical stability, and its compatibility with highly sensitive detection methodologies such as methylation-specific polymerase chain reaction and bisulfite sequencing [90].

A significant body of research has validated the diagnostic utility of tissue-based methylation assays employing specific gene panels. For instance, a well-characterized panel featuring markers like MINT25, LOX, and ADAMTS9 has demonstrated high sensitivity and specificity in distinguishing malignant gastric tissue from both adjacent normal mucosa and benign lesions. The diagnostic power of such panels lies not only in their ability to confirm the presence of established carcinoma but also in their potential to detect molecular abnormalities in precancerous states, such as high-grade dysplasia [91]. This capability suggests a role for methylation markers in risk stratification, potentially identifying individuals with pre-malignant conditions who would benefit from intensified surveillance or early intervention, thereby shifting the clinical management paradigm towards a more preventative approach [92].

Despite the promising diagnostic performance of these methylation markers, several technical limitations inherent to the detection methodologies must be acknowledged to ensure accurate clinical interpretation.

Bisulfite conversion artifacts. Bisulfite sequencing, while considered the gold standard for DNA methylation analysis, is associated with well-documented technical artifacts. First, incomplete conversion of unmethylated cytosines to uracil represents a major concern, with reported conversion efficiencies ranging from 95–98% rather than the theoretical 99.5–99.7% [93]. Even minimal residual unconverted cytosines (1–5%) can be misinterpreted as methylated CpG sites, leading to false-positive methylation calls, particularly in GC-rich regions such as CpG islands, where secondary structures may impede complete conversion [94]. Second, bisulfite treatment inherently causes substantial DNA degradation. The harsh chemical conditions induce depyrimidination, resulting in DNA fragmentation and biased recovery of intact fragments. This degradation is particularly problematic for low-input samples and leads to uneven genome coverage, with systematic underrepresentation of GC-rich regions [95].

Methylation-specific PCR bias. Methylation-specific PCR, widely used for its simplicity and sensitivity, carries inherent design-related biases. As noted in technical guidelines, MSP primers must contain at least one CpG site, ideally positioned at the 3′-end to maximize discrimination between methylated and unmethylated templates [96]. However, this design constraint limits the ability to assess single CpG methylation status within CpG islands. Furthermore, the requirement that both methylated and unmethylated primer pairs target the same CpG sites while maintaining similar annealing temperatures often proves challenging, potentially introducing amplification bias [97]. Importantly, MSP is fundamentally qualitative or semi-quantitative at best; the exponential nature of PCR amplification can exaggerate minor differences in initial template abundance, leading to overestimation of methylation levels when the signal-to-noise ratio is low. These technical considerations underscore the need for rigorous validation of diagnostic methylation assays before clinical implementation.

### 4.2 Circulating Tumor DNA

To overcome the limitations of tissue-based approaches, the field has pivoted toward liquid biopsy, a revolutionary paradigm centered on the analysis of circulating tumor DNA. ctDNA comprises short fragments of tumor-derived DNA shed into the bloodstream through processes such as apoptosis, necrosis, and active secretion. It carries the complete molecular signature of the tumor, including its unique epigenetic landscape. The analysis of ctDNA methylation patterns offers distinct advantages over mutation-based liquid biopsies [98]. Aberrant promoter methylation is an early and nearly universal event in gastric carcinogenesis, affecting a high proportion of tumor cells. This makes methylated ctDNA signals potentially more abundant and homogeneous than low-frequency somatic mutations, which can be challenging to detect against the background of wild-type DNA, especially in early-stage disease or cases with low tumor burden.

Several specific gene methylation markers have been extensively validated for ctDNA-based gastric cancer detection. The Septin 9 (*SEPT9*) gene is one of the most well-characterized cfDNA methylation biomarkers; while clinically approved for colorectal cancer screening, accumulating evidence supports its diagnostic utility in gastric cancer, with reported sensitivity of 70–80% and specificity exceeding 90%. Another promising panel combines *RNF180* and RPRM, which has demonstrated superior diagnostic performance in plasma and gastric juice samples, with a combined sensitivity of approximately 70% and specificity of 90%. The *CDH1* (E-cadherin) gene, frequently methylated in diffuse-type gastric cancer, represents a valuable marker for detecting this aggressive histological subtype through cfDNA analysis. Classical tumor suppressor genes including p16/*TYMS*, *RUNX3*, and *RASSF1A* have also been successfully detected in cfDNA from gastric cancer patients, with methylation frequencies ranging from 30–60% depending on the gene and tumor stage [68,69,71]. For enhanced diagnostic accuracy, multi-gene methylation panels have been developed; for instance, the panel comprising MINT25, LOX, and ADAMTS9 has shown high sensitivity and specificity (>85%) in discriminating gastric cancer from benign gastric conditions.

**Figure 3:**
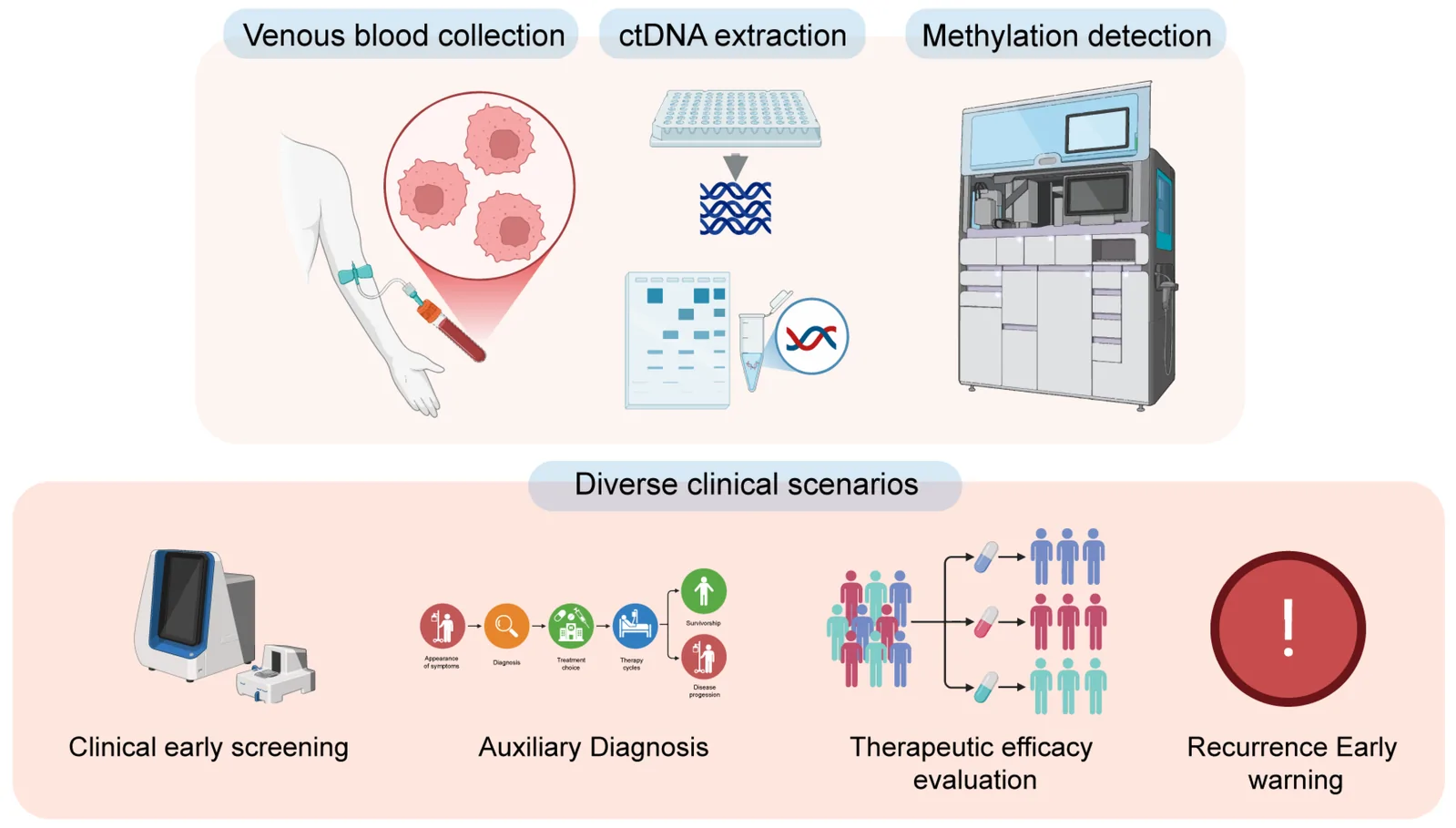
Application Workflow of ctDNA Methylation-Based Liquid Biopsy in Gastric Cancer Management. Schematic illustration of the liquid biopsy workflow using circulating tumor DNA (ctDNA) methylation analysis for gastric cancer. The process begins with venous blood collection from the patient, followed by ctDNA extraction and subsequent methylation profiling. The resulting methylation data are applied across diverse clinical scenarios, including early screening, auxiliary diagnosis, minimal residual disease (MRD) monitoring, therapeutic efficacy evaluation, and early recurrence warning. This non-invasive approach enables dynamic and personalized management throughout the gastric cancer care continuum. Original figure drawn independently using Adobe Illustrator (Version 29.7, Adobe Inc., USA, official website: https://www.adobe.com/).

Technological advancements in ultra-deep, targeted bisulfite sequencing and digital droplet PCR have enabled the sensitive and specific detection of these trace methylation signals in plasma. This has unlocked a transformative, non-invasive tool with applications across the entire clinical spectrum of gastric cancer management. In the setting of early detection and screening, panels of highly specific gastric cancer methylation markers analyzed from a simple blood draw hold promise as a cost-effective and accessible triage test. Such a test could identify asymptomatic, high-risk individuals in the general population who should then proceed to confirmatory endoscopic evaluation, thereby increasing screening efficiency and compliance. Following curative-intent surgery, the presence or absence of methylated ctDNA serves as a powerful tool for minimal residual disease monitoring. A positive post-operative ctDNA signal can indicate residual microscopic disease and predict imminent clinical relapse months before it becomes radiographically apparent, allowing for early intervention with adjuvant therapy. For patients with advanced disease undergoing systemic treatment, serial quantitative monitoring of specific methylated ctDNA markers provides a dynamic, real-time barometer of therapeutic efficacy. A rapid decline in ctDNA levels may indicate a favorable response, while its persistence or rise can signal primary or acquired resistance, enabling timely therapeutic adjustments. Finally, during surveillance, periodic ctDNA testing acts as a molecular radar for recurrence, offering a highly sensitive method to detect disease resurgence. By converting tumor DNA into a circulating, repeatedly accessible informant, ctDNA methylation analysis is fundamentally shifting gastric cancer care from a reactive, imaging-based model to a proactive, molecularly guided precision oncology approach [99] (Fig. 3).

Methodological challenges in low-frequency allele detection. While ctDNA methylation analysis offers tremendous promise for non-invasive cancer management, the detection of low-frequency methylated alleles in circulation presents significant technical challenges with major clinical implications, particularly for minimal residual disease monitoring. Standard bisulfite sequencing approaches may fail to distinguish true biological methylation heterogeneity from technical artifacts such as incomplete conversion or PCR errors. As demonstrated by Warnecke et al. [97], PCR-induced mutations occur at frequencies of approximately 0.1–1% per base, which can confound the detection of rare methylated alleles.

Moreover, recent evidence from mitochondrial DNA methylation studies highlights that non-CpG methylation can bias bisulfite PCR toward amplification of low or unmethylated templates [100]. While this finding pertains primarily to mitochondrial DNA, it raises broader concerns about the specificity of primer design across different sequence contexts.

In the context of circulating tumor DNA analysis, the extremely low abundance of tumor-derived fragments (often <0.1% of total cell-free DNA) exacerbates these technical challenges. Digital droplet PCR and ultra-deep bisulfite sequencing have been developed to address these limitations, achieving detection limits as low as 0.01% for methylated alleles [81]. However, these approaches require rigorous validation and quality control measures, including the use of unique molecular identifiers to correct for PCR duplication bias, spike-in controls, and replicate analyses, to ensure that detected signals reflect true biological methylation rather than technical noise. Establishing limit-of-blank and limit-of-detection parameters for each assay is essential before clinical deployment.

### 4.3 Prognosis and Recurrence Monitoring

#### 4.3.1 Correlation of Methylation Signatures with Tumor Stage, Lauren Classification, and Survival

Beyond its diagnostic utility, the DNA methylome provides a rich source of prognostic information, offering molecular insights that refine risk stratification beyond conventional clinicopathological parameters. The association between specific methylation signatures and established prognostic factors, such as tumor stage and Lauren classification, is well-documented. Tumors exhibiting a high degree of promoter hypermethylation, particularly within genes governing critical pathways like cell cycle control and DNA repair, frequently correlate with more advanced pathological stages and aggressive phenotypes. For instance, the Lauren intestinal subtype, often linked to a multi-step carcinogenesis model, may show distinct methylation patterns, such as frequent *TYMS* silencing, associated with its specific biological behavior [101]. More importantly, numerous studies have established that specific methylation profiles serve as independent predictors of overall and disease-free survival. A global assessment, like the CpG Island Methylator Phenotype, or the methylation status of single genes like *RUNX3* or *RASSF1A*, can stratify patients into groups with significantly different clinical outcomes, identifying those with a higher likelihood of disease progression and mortality [102].

#### 4.3.2 Methylation Biomarkers for Predicting Postoperative Recurrence and Metastatic Risk

This prognostic power translates directly into a crucial clinical application: the prediction of postoperative recurrence and metastatic risk. Following curative resection, the primary clinical challenge is identifying which patients harbor occult microscopic disease destined to relapse. Methylation markers detected in postoperative tissue or, more dynamically, in plasma ctDNA, have emerged as powerful tools for this purpose. The presence of tumor-specific methylation signals in post-operative blood samples, a state known as molecular residual disease, is a potent predictor of subsequent clinical recurrence. Patients with detectable methylated ctDNA after surgery exhibit a dramatically higher risk of relapse compared to those with undetectable MRD. Furthermore, the specific pattern of methylation can offer clues about the nature of the relapse; for example, methylation signatures associated with epithelial-mesenchymal transition may portend a higher risk of distant metastasis. This capability for early, post-operative risk assessment enables a more personalized approach to adjuvant therapy. High-risk patients identified by methylation markers could be candidates for more intensive treatment or closer surveillance, while low-risk patients might be spared unnecessary adjuvant chemotherapy, aligning with the goals of precision oncology to maximize efficacy while minimizing toxicity [9].

#### 4.3.3 Methylation Biomarkers for Dynamic Therapeutic Response Monitoring

The static assessment of prognosis naturally extends into the dynamic monitoring of therapeutic efficacy, representing a paradigm shift towards real-time, personalized cancer management. Methylation biomarkers, particularly when analyzed in serial liquid biopsies, offer an unprecedented window into the molecular response of a tumor to treatment, overcoming the limitations of delayed and often imprecise radiographic evaluations.

During systemic therapy for advanced gastric cancer, whether with chemotherapy, targeted agents, or immunotherapy, the quantitative changes in circulating tumor DNA methylation levels serve as a highly sensitive pharmacodynamic readout. A rapid and significant decrease in the concentration of tumor-specific methylated ctDNA fragments following treatment initiation often correlates with a favorable tumor response and improved progression-free survival. This molecular response can be detected within days or weeks, far earlier than any shrinkage observable on computed tomography scans. Conversely, the persistence of or an increase in methylated ctDNA signals, even in the context of seemingly stable imaging findings, may indicate primary resistance or the emergence of clonal populations resistant to therapy [9]. This early warning allows clinicians to consider altering the treatment strategy before clinical progression occurs, potentially improving outcomes [103].

This application is especially promising in the context of immunotherapy. The efficacy of immune checkpoint inhibitors is known to be influenced by the tumor’s epigenetic state. For instance, hypomethylating agents can sensitize tumors to immunotherapy by upregulating antigen presentation and interferon signaling. Monitoring the methylation status of genes involved in these pathways within ctDNA could provide insights into the dynamic remodeling of the tumor immune microenvironment during treatment and help predict or confirm response to immunotherapeutic regimens [104]. Thus, by transforming ctDNA into a “liquid molecular thermometer,” DNA methylation analysis enables a shift from reactive, scan-based decision-making to a proactive, biomarker-guided approach, optimizing therapeutic sequences and improving the personalization of care for patients with gastric cancer.

### 4.4 Prediction of Therapeutic Response

The ultimate goal of biomarker development in oncology is to guide therapeutic decisions, moving beyond generic protocols toward truly personalized medicine. DNA methylation profiles hold significant promise for predicting a tumor’s inherent sensitivity or resistance to specific treatments, thereby enabling the selection of the most effective therapy from the outset [15].

#### 4.4.1 Predicting Chemotherapy Sensitivity

Chemotherapy remains a cornerstone of treatment for gastric cancer, yet responses are heterogeneous. The methylation status of specific genes involved in drug metabolism and DNA repair pathways can serve as predictive biomarkers for chemotherapy efficacy. A prime example is the prediction of sensitivity to fluoropyrimidine-based regimens, such as 5-fluorouracil. The enzyme thymidylate synthase is a critical target of 5-FU, and its expression levels are inversely correlated with treatment response. The promoter methylation status of the *TYMS* gene, which encodes thymidylate synthase, has been investigated as a regulator of its expression [105]. Hypomethylation of the *TYMS* promoter, leading to high enzyme expression, is associated with 5-FU resistance. Conversely, hypermethylation and subsequent low expression may predict better sensitivity. Similarly, the methylation status of the DPYD gene, involved in 5-FU catabolism, can influence drug toxicity and efficacy. Beyond fluoropyrimidines, methylation of the *MGMT* promoter, which leads to silencing of the DNA repair protein O6-methylguanine-DNA methyltransferase, predicts sensitivity to alkylating agents like temozolomide by impairing the cell’s ability to repair drug-induced DNA damage [105]. Integrating these methylation markers into clinical decision-making could help stratify patients into those most likely to benefit from standard chemotherapy and those who should be considered for alternative regimens, thereby avoiding ineffective treatment and its associated toxicity [106].

#### 4.4.2 Predicting Response to Immune Checkpoint Inhibitors

The advent of immunotherapy has revolutionized cancer treatment, but reliable biomarkers for patient selection are crucial. DNA methylation plays a dual role in modulating the tumor immune microenvironment and has emerged as a key predictor of response to immune checkpoint inhibitors. The most established link is through the microsatellite instability-high phenotype, which is frequently caused by epigenetic silencing of the *MLH1* mismatch repair gene via promoter hypermethylation. MSI-H tumors harbor a high tumor mutational burden and neoantigen load, making them highly responsive to PD-1 blockade [84]. Therefore, detecting *MLH1* promoter methylation in tumor tissue serves as a robust predictive biomarker for immunotherapy benefit in gastric cancer [107].

Beyond MSI-H, the broader CpG Island Methylator Phenotype, especially the extreme CIMP observed in EBV-positive gastric cancers, is also associated with favorable responses to immunotherapy. The widespread promoter hypermethylation in CIMP-high tumors can lead to transcriptional silencing of multiple genes, but it also frequently induces the expression of endogenous retroviral elements, creating a state of “viral mimicry.” This triggers a constitutive interferon response and enhances tumor immunogenicity, making these tumors more visible and vulnerable to the immune system. Consequently, a CIMP-high methylation signature, detectable through specific gene panels, may identify a broader subset of patients beyond those with MSI-H who are likely to respond to immune checkpoint inhibitors [108]. Thus, DNA methylation analysis provides a powerful framework for identifying gastric cancer patients who stand to gain the most from immunotherapy, encompassing both the well-defined MSI-H pathway and the emerging CIMP-associated mechanisms of immune sensitization (Table 2).

**Table 2:** DNA methylation biomarkers with clinical translation potential in gastric cancer.

Biomarker/Gene Panel	Sample Type	Clinical Application	Detection Platform/Assay Modality	Performance Characteristics/Key Information	Research Stage	Reference
Septin 9 (*SEPT9*)	Plasma	Diagnosis/Early Detection	qMSP; commercial assay (Epi proColon)	Sensitivity ~70–80%, specificity ~90–95%. Clinically approved for other cancers (e.g., colorectal).	Clinical Validation/Early Clinical Use	[109]
*RNF180*/RPRML	Plasma, Gastric Juice	Diagnosis	qMSP	Combined detection in plasma: sensitivity ~70%, specificity ~90%. Performance is superior in gastric juice.	Clinical Validation	[92]
MINT25, LOX, ADAMTS9 Panel	Tissue, Gastric Juice	Diagnosis	MSP; bisulfite sequencing	In tissue: sensitivity and specificity > 85%. Effective in discriminating GC from benign conditions in gastric juice.	Clinical Validation	[56]
ctDNA Multi-gene Methylation Panel	Plasma	MRD Monitoring, Response Evaluation, Relapse Warning	Targeted bisulfite sequencing; ddPCR	Ultra-high sensitivity (<0.1% tumor fraction). Dynamic changes strongly correlate with clinical outcome.	Clinical Research/Translational Validation	[110]
*MLH1*	Tissue	Diagnosis, Prognosis, Prediction	MSP; qMSP	Methylation causes the MSI-H phenotype; primary cause of sporadic Lynch syndrome cases. Strong predictive biomarker for immune checkpoint inhibitor response.	Clinical Use (for MSI/dMMR testing)	[71]
EBV Methylation Signature	Tissue	Prognosis, Prediction	Bisulfite sequencing; targeted methylation panel	Associated with EBV-positive GC. These tumors have distinct clinicopathological features and high PD-L1 expression, suggesting potential heightened sensitivity to immunotherapy.	Clinical Research/Translational Validation	[111]
*CDH1* (E-cadherin)	Tissue	Diagnosis, Prognosis	MSP; qMSP	High methylation frequency in diffuse-type GC. Associated with tumor invasiveness, poor prognosis, and hereditary diffuse gastric cancer.	Clinical Research	[70]
*RUNX3*, *RASSF1A*, DAPK Prognostic Panel	Tissue	Prognosis	MSP; qMSP	Methylation status of a multi-gene panel can serve as an independent prognostic risk stratification factor, significantly correlating with overall and disease-free survival.	Clinical Validation	[112]
*TYMS*/DPYD Methylation Status	Tissue	Prediction	qMSP; bisulfite sequencing	Predicts potential sensitivity to fluoropyrimidine-based chemotherapy (low *TYMS* expression) or toxicity risk (*DPYD*).	Exploratory/Clinical Research	[105]
MGMT	Tissue	Prediction	MSP; qMSP	Promoter methylation leads to gene silencing and may predict sensitivity to alkylating agents (e.g., temozolomide).	Exploratory/Clinical Research	[71]

**Note: MSP**, Methylation-Specific PCR, **qMSP**, quantitative Methylation-Specific PCR; *RNF180*, ring finger protein 180; **RPRM**, Reprimo, TP53 Dependent G2 Arrest Mediator Homolog; **MINT25**, Methylated IN Tumor 25; **ADAMTS9**, ADAM Metallopeptidase with Thrombospondin Type 1 Motif 9; ***MLH1***, MutL Homolog 1; **ctDNA**, circulating tumor DNA; **EBV**, Epstein-Barr virus; ***CDH1***, Cadherin 1; ***RUNX3***, Runt-Related Transcription Factor 3; ***RASSF1A***, Ras Association Domain Family Member 1A; **DAPK**, Death-Associated Protein Kinase; ***TYMS*:** Thymidylate Synthase; ***MGMT***, O^6^-Methylguanine-DNA Methyltransferase; **ddPCR**, Droplet Digital Polymerase Chain Reaction; **GC:** Gastric Cancer; **PD-L1**, programmed death-ligand 1.

## 5 Therapeutic Strategies Targeting DNA Methylation

The central role of DNA methylation in driving gastric carcinogenesis makes it an exceptionally attractive therapeutic target. Strategies targeting DNA methylation primarily focus on reversing aberrant epigenetic silencing, aiming to restore the function of suppressed tumor suppressor genes and re-establish normal gene expression programs. These approaches range from direct pharmacological inhibition of the methylation machinery to rational combinations with existing therapies, representing a paradigm shift in gastric cancer treatment that moves beyond targeting genetic mutations alone [113].

### 5.1 Hypomethylating Agents

Hypomethylating agents, also known as demethylating drugs, constitute the first generation of epigenetic therapies. Their primary mechanism of action is to inhibit DNA methyltransferases, the enzymes responsible for establishing and maintaining DNA methylation patterns [114].

#### 5.1.1 Nucleoside Analogues

The most clinically advanced HMAs (HMAs, Hypomethylating Agents) are nucleoside analogues, primarily azacitidine and its deoxy derivative, decitabine [115]. These drugs are structurally similar to cytidine and are incorporated into newly synthesized DNA during cell division. Once incorporated, they form a covalent, irreversible complex with DNA methyltransferases, effectively trapping and depleting the cellular pool of these enzymes. During subsequent rounds of DNA replication in the absence of functional DNMTs, methylation marks cannot be faithfully copied to the daughter strands, leading to passive, genome-wide DNA demethylation. This process is not random but preferentially affects regions with high replication rates, such as actively dividing cancer cells. The consequent global reduction in methylation, particularly at the hypermethylated promoters of tumor suppressor genes, can lead to their transcriptional reactivation. This reactivation can restore critical cellular functions like cell cycle control, apoptosis, and differentiation, thereby inhibiting tumor growth and survival. It is important to note that the effects are not instantaneous and require cell division, explaining the delayed clinical response often observed with these agents [116].

#### 5.1.2 Preclinical Models and Early Clinical Trials

Preclinical studies in gastric cancer cell lines and xenograft models have provided compelling proof-of-concept for HMAs. Treatment with decitabine or azacitidine has been shown to induce global DNA hypomethylation, reactivate silenced tumor suppressor genes such as p16/*TYMS*, *RASSF1A*, and E-cadherin, and exert potent anti-proliferative and pro-apoptotic effects. Importantly, these drugs have demonstrated efficacy even in chemoresistant models, suggesting their potential to overcome drug resistance [117].

Despite these promising preclinical results, the translation of HMAs into effective single-agent therapies for solid tumors, including gastric cancer, has been challenging, as reflected in early-phase clinical trials. The limitations are multifactorial. First, pharmacokinetics and toxicity: As systemic agents, HMAs affect all rapidly dividing cells, leading to dose-limiting hematological toxicities such as neutropenia and thrombocytopenia, which mirror their use in myeloid malignancies. Achieving sufficient drug concentrations in solid tumors without intolerable side effects is difficult. Second, lack of specificity: The genome-wide demethylation induced by HMAs is a double-edged sword. While it may reactivate tumor suppressors, it can also potentially demethylate and activate oncogenes or parasitic genomic elements, with unpredictable consequences. Third, tumor heterogeneity and adaptive resistance: Epigenetic states are dynamic [118]. Tumors may develop resistance through mechanisms independent of DNA methylation, or subpopulations of cancer stem cells with different epigenetic landscapes may survive treatment. Fourth, biomarker deficiency: Most trials have lacked robust biomarkers to identify the subset of patients most likely to respond, such as those with a high degree of promoter hypermethylation. Consequently, while early trials have shown occasional responses and disease stabilization, consistent and durable single-agent activity in unselected gastric cancer populations has not been established. These experiences have underscored that the future of epigenetic therapy likely lies not in monotherapy but in rational combination strategies. This perspective is further supported by the understanding that DNA methylation operates within a broader chromatin context; stable gene silencing often involves coordinated repressive histone modifications that may not be fully reversed by hypomethylating agents alone. Thus, combination approaches targeting both DNA methylation and histone modifications hold particular promise.

### 5.2 Novel Combination Therapeutic Paradigms

The limitations of single-agent HMAs have spurred the development of innovative combination strategies. The rationale is to exploit the ability of demethylating agents to “re-sensitize” cancer cells to other treatments by reversing specific epigenetic blocks.

#### 5.2.1 Combination with Chemotherapy or Targeted Therapy: Reversing Resistance

A key mechanism of resistance to conventional chemotherapy and targeted agents is the epigenetic silencing of genes critical for drug sensitivity. HMAs can be used to reverse this silencing, thereby restoring therapeutic vulnerability. For instance, resistance to 5-fluorouracil can be associated with the overexpression of thymidylate synthase. As the *TYMS* gene promoter can be regulated by methylation, pretreatment with an HMA might lower TS expression and re-sensitize cells to 5-FU. Similarly, resistance to platinum drugs can involve the silencing of DNA repair genes; their reactivation could theoretically restore sensitivity, although the effect might be complex [119]. In the realm of targeted therapy, resistance to trastuzumab in HER2-positive gastric cancer has been linked to epigenetic silencing of PTEN. Preclinical evidence suggests that decitabine can restore PTEN expression and re-establish sensitivity to HER2 inhibition [120]. These combinations aim to use low, non-cytotoxic doses of HMAs as “epigenetic primers” to modulate the expression of specific pathways, followed by standard cytotoxic or targeted agents. This approach could broaden the efficacy of existing drugs and overcome acquired resistance, offering a new lease on life for established therapies [121].

#### 5.2.2 Combination with Immunotherapy: Enhancing Tumor Immunogenicity

The most promising and mechanistically sophisticated combination paradigm pairs HMAs with immune checkpoint inhibitors. This synergy is rooted in the ability of DNA demethylation to fundamentally reshape the tumor-immune microenvironment. HMAs can enhance tumor immunogenicity through several interconnected mechanisms. First, viral mimicry: By inducing global DNA hypomethylation, HMAs can reactivate dormant endogenous retroviral elements embedded in the human genome. The transcription and translation of these ERV sequences generate double-stranded RNA and viral-like proteins, which are detected by the cell as a viral infection. This triggers a potent innate immune response characterized by the production of type I and III interferons. This “viral mimicry” state creates a pro-inflammatory tumor microenvironment, recruits immune cells, and upregulates antigen presentation machinery on tumor cells. Second, antigen upregulation: HMAs can directly reactivate genes encoding cancer-testis antigens and other tumor-associated antigens, increasing the repertoire of targets available for immune recognition. Third, reversal of immune silencing: Tumors often epigenetically silence genes involved in antigen processing and presentation or in the IFN signaling pathway itself [122]. Demethylation can restore these critical circuits, making tumors more visible to T cells [123].

When this HMA-induced “hot” immunological state is combined with PD-1/PD-L1 checkpoint blockade, the effects are synergistic. The activated T cells, recruited and primed by the HMA, are now unleashed from inhibitory signals by the immunotherapy. This combination has shown remarkable success in other CIMP-high malignancies like myelodysplastic syndrome and is the subject of intense investigation in gastric cancer. Preclinical models of gastric cancer confirm that decitabine upregulates PD-L1 and enhances response to anti-PD-1 therapy. Early-phase clinical trials are actively exploring this combination, particularly in biologically relevant subgroups such as EBV-positive (extreme CIMP) and MSI-H gastric cancers, where the epigenetic and immune dysregulation are intrinsically linked [124]. This strategy represents the frontier of epigenetic-immuno-oncology, aiming to convert immunologically “cold” gastric tumors into “hot” ones susceptible to immune-mediated destruction.

### 5.3 Current Challenges

While the therapeutic potential of targeting DNA methylation is immense, significant challenges impede its seamless translation into routine clinical practice for gastric cancer. A primary and formidable obstacle is the lack of tissue specificity inherent to current hypomethylating agents, leading to dose-limiting systemic toxicity. As nucleoside analogues, drugs like azacitidine and decitabine are incorporated into the DNA of all rapidly dividing cells. This results in profound on-target, off-tumor effects, most notably severe myelosuppression including neutropenia and thrombocytopenia, which mirrors their established toxicity profile in hematological malignancies [125]. Achieving therapeutic drug concentrations within solid tumors without inducing intolerable hematological toxicity remains a delicate and often unsuccessful balance, limiting the dose intensity that can be safely administered. Compounding this issue is the unresolved question of the optimal therapeutic regimen. The appropriate dose, schedule, and duration of treatment for solid tumors are empirically derived and poorly defined. Should HMAs be administered in low, prolonged “epigenetic priming” doses or in higher, pulsed regimens? What is the ideal sequence and timing when combining them with chemotherapy or immunotherapy? For instance, preclinical data often suggest that a period of HMA pretreatment is necessary to alter the epigenetic landscape before immune cells can effectively engage, but this paradigm requires rigorous clinical validation [126]. Finally, and critically, the field suffers from a pronounced lack of validated predictive biomarkers. Administering these agents to an unselected gastric cancer population is likely to yield low response rates and expose many patients to unnecessary toxicity. There is an urgent need to identify which patients harbor tumors that are “addicted” to or highly dependent on specific epigenetic silencing for survival, such as those with a high CpG Island Methylator Phenotype or specific patterns of tumor suppressor gene hypermethylation [127]. The development of robust biomarkers, potentially detectable in blood-based liquid biopsies, to predict which patients will derive clinical benefit is essential for the rational and cost-effective application of these therapies. Overcoming these intertwined challenges of toxicity, regimen optimization, and patient selection is the crucial next step required to unlock the full clinical potential of epigenetic therapy in gastric cancer [128].

## 6 Conclusion

The journey of DNA methylation in gastric cancer, from a fundamental biological mechanism to a cornerstone of clinical innovation, illustrates a paradigm shift in oncology. Its dual role as both a driver of carcinogenesis and a reservoir of biomarkers underscores its central position in the disease’s molecular architecture. The epigenetic landscape, reshaped by factors like *H. pylori* infection, creates a permissive field for malignancy, silencing tumor suppressors and destabilizing the genome. This very stability and specificity of methylation marks, however, render them ideal clinical tools. The advent of liquid biopsy, detecting tumor-derived methylated DNA in blood, is revolutionizing patient management by enabling non-invasive diagnosis, real-time monitoring of treatment response, and early detection of relapse long before clinical symptoms arise [129].

Translating this profound understanding into tangible patient benefit is the critical next frontier. This requires bridging the gap between laboratory discovery and bedside application. For biomarkers, robust clinical trials must validate multi-gene methylation panels, moving them from research tools to standardized diagnostics that can guide everyday therapeutic decisions. For treatment, the challenge is to evolve beyond the limitations of first-generation drugs. Future strategies will likely hinge on developing more selective epigenetic modulators and designing intelligent combination therapies, particularly with immunotherapy, where demethylating agents can effectively “prime” the tumor immune microenvironment. Success in these endeavors fundamentally depends on identifying the right patients through predictive biomarkers, ensuring that these powerful interventions are delivered to those most likely to respond.

The ultimate promise lies in a fully integrated model of precision care. Imagine a future where a simple blood test profiles an individual’s epigenetic risk, enabling personalized prevention. At diagnosis, a tumor’s methylome is instantly decoded, guiding prognosis and predicting the optimal therapeutic path. For advanced disease, epigenetic therapies are strategically deployed to resensitize tumors to treatment and unleash the immune system. By mastering the epigenetic code of gastric cancer, we are not merely adding another tool to our arsenal; we are fundamentally redefining the approach to the disease, aiming to transform it from a lethal threat into a manageable condition through earlier interception, sharper classification, and smarter, more effective treatment [130].

## Data Availability

This article is a comprehensive review and does not involve the generation of new experimental or clinical data. All data analyzed or discussed in this manuscript are derived from previously published studies, which have been appropriately cited in the reference list. No new datasets were created or analyzed during the current study. Therefore, data sharing is not applicable to this article.

## Abbreviation

DNMTs	DNA methyltransferases
CpG	cytosine-phosphate-guanine
EBV	Epstein-Barr virus
CIMP	CpG Island Methylator Phenotype
MSI	microsatellite instability
LINE-1	Long Interspersed Nuclear Element 1
HMA	hypomethylating agents
ctDNA	circulating tumor
MSI-H	microsatellite instability-high
MRD	minimal residual disease
5-FU	5-fluorouracil
PD-L1	programmed death-ligand 1

## References

[1] Machlowska J , Baj J , Sitarz M , Maciejewski R , Sitarz R . Gastric cancer: Epidemiology, risk factors, classification, genomic characteristics and treatment strategies. Int J Mol Sci. 2020; 21( 11): 4012. doi:10.3390/ijms21114012. 32512697 PMC7312039

[2] Ubaid S , Kushwaha R , Kashif M , Singh V . Comprehensive analysis of oncogenic determinants across tumor types via multi-omics integration. Cancer Genet. 2025; 298: 44– 62. doi:10.1016/j.cancergen.2025.08.010. 40914134

[3] Matsusaka K . DNA methylation in gastric cancer, related to *Helicobacter pylori* and Epstein-Barr virus. World J Gastroenterol. 2014; 20( 14): 3916. doi:10.3748/wjg.v20.i14.3916. 24744581 PMC3983447

[4] Christodoulidis G , Koumarelas KE , Kouliou MN , Thodou E , Samara M . Gastric cancer in the era of epigenetics. Int J Mol Sci. 2024; 25( 6): 3381. doi:10.3390/ijms25063381. 38542354 PMC10970362

[5] Yamada H , Abe S , Charvat H , Ando T , Maeda M , Murakami K , et al. Precision risk stratification of primary gastric cancer after eradication of Helicobacter pylori by a DNA methylation marker: A multicentre prospective study. Gut. 2025; 74( 9): 1410– 8. doi:10.1136/gutjnl-2025-335039. 40240063 PMC12418579

[6] Huang A , Guo DZ , Su ZX , Zhong YS , Liu L , Xiong ZG , et al. GUIDE: A prospective cohort study for blood-based early detection of gastrointestinal cancers using targeted DNA methylation and fragmentomics sequencing. Mol Cancer. 2025; 24: 163. doi:10.1186/s12943-025-02367-x. 40468355 PMC12139136

[7] Lee YC . Single-biopsy epigenetic profiling reveals persistent DNA methylation and gastric cancer risk after *Helicobacter pylori* eradication. Gut. 2026; 75( 1): 2– 4. doi:10.1136/gutjnl-2025-335329. 40268348

[8] Wang Y , Jin L , He W , Peng J , Yin K , Liu X , et al. Plasma cell-free DNA methylation markers for detection and prognosis of gastric cancer: A case-control study. Chin J Cancer Res. 2025; 37( 5): 850– 64. doi:10.21147/j.issn.1000-9604.2025.05.14. PMC12603634 41229986

[9] Huang HY , Lan J , Zhuang W . Role of circulating tumor DNA methylation in gastric cancer initiation and progression: A comprehensive review. World J Gastrointest Oncol. 2025; 17( 8): 107412. doi:10.4251/wjgo.v17.i8.107412. 40837757 PMC12362539

[10] Newell ME , Babbrah A , Aravindan A , Rathnam R , Halden RU . DNA methylation in urine and feces indicative of eight major human cancer types globally. Life. 2025; 15( 3): 482. doi:10.3390/life15030482. 40141826 PMC11943902

[11] Maeda M , Moro H , Ushijima T . Mechanisms for the induction of gastric cancer by *Helicobacter pylori* infection: Aberrant DNA methylation pathway. Gastric Cancer. 2017; 20( 1): 8– 15. doi:10.1007/s10120-016-0650-0. 27718135

[12] Dede S , Şakalar Ç , Yılmaz B , Çubukcı G , Acar M , Yavuz A , et al. SFRP2 and RPRM as methylation based serum biomarkers for the detection of gastric cancer. Discov Oncol. 2025; 16( 1): 1606. doi:10.1007/s12672-025-03472-5. 40849852 PMC12375522

[13] Gu J , Wu Y , Tao W , Xin J , Liu H , Gong W , et al. An integrative multi-omics study to identify candidate DNA methylation biomarkers associated with gastric cancer prognosis. Arch Toxicol. 2025; 99( 10): 4067– 80. doi:10.1007/s00204-025-04118-9. 40616607

[14] Dong W , Lau CH , Li J , Huang Z , Li J , Wu W , et al. Pan-cancer methylation analysis of circulating cell-free DNA. Cancer Genet. 2025; 296: 182– 95. doi:10.1016/j.cancergen.2025.07.014. 40737714

[15] Tang W , Zhu Z , Wang Z , Li G , Lin Q , Cai S , et al. DNA methylation profiles predicting response to anti-PD-1-based treatment in patients with advanced gastric cancer. BMC Med. 2025; 23( 1): 529. doi:10.1186/s12916-025-04357-8. 41034874 PMC12487477

[16] Kalra A , Meltzer SJ . The role of DNA methylation in gastrointestinal disease: An expanded review of malignant and nonmalignant gastrointestinal diseases. Gastroenterology. 2025; 168( 2): 245– 66. doi:10.1053/j.gastro.2024.07.001. 38971197 PMC11698954

[17] Mano Y , Matsusaka K , Seki M , Kita K , Fukuyo M , Rahmutulla B , et al. DNA-hypermethylated human gastric cancer circumvents apoptosis in the absence of *TP53* mutation. J Pathol. 2025; 267( 4): 435– 49. doi:10.1002/path.6480. 41065300

[18] Jiang H , Hou R , Wu X , Ding K , Zhu Y , Wang H , et al. DNA-methylation-mediated silencing of lncRNA PP7080 promotes the progression of gastric cancer by regulating ANKRD1 expression. Transl Oncol. 2025; 59: 102440. doi:10.1016/j.tranon.2025.102440. 40516143 PMC12206047

[19] Iden CR , Mustafa SM , Øgaard N , Henriksen T , Jensen SØ , Ahlborn LB , et al. Circulating tumor DNA predicts recurrence and survival in patients with resectable gastric and gastroesophageal junction cancer. Gastric Cancer. 2025; 28( 1): 83– 95. doi:10.1007/s10120-024-01556-9. 39369091 PMC11706848

[20] Kaur H , Lin LH , Kolin DL , Pinto A , Parra-Herran C , Catherwood M , et al. Primary endometrial gastric (gastrointestinal)-type mucinous adenocarcinoma: A detailed clinicopathologic and molecular analysis of 27 cases. Am J Surg Pathol. 2025; 49( 6): 564– 77. doi:10.1097/PAS.0000000000002382. 40066786

[21] Su C , Lin Z , Ye Z , Liang J , Yu R , Wan Z , et al. Development of a prognostic model for early-stage gastric cancer-related DNA methylation-driven genes and analysis of immune landscape. Front Mol Biosci. 2024; 11: 1455890. doi:10.3389/fmolb.2024.1455890. 39575189 PMC11579923

[22] Hu Y , Liu S , Cui C , Liu X , Li H , Liu H , et al. Enhanced HER2 status detection in breast and gastric cancers using surrogate DNA methylation markers. IUBMB Life. 2025; 77( 2): e70004. doi:10.1002/iub.70004. 39988770

[23] Yin Y , Wang B , Yang M , Liu W , Wang A , Lu Z , et al. Comprehensive analysis of MAGE-A10 in pan-cancer and its validation in gastric cancer. Sci Rep. 2025; 15: 32077. doi:10.1038/s41598-025-17987-y. 40890425 PMC12402061

[24] Ansari S , Ahmed N . Pathogenicity of *Helicobacter pylori*-associated gastric cancer. World J Clin Oncol. 2025; 16( 12): 110909. doi:10.5306/wjco.v16.i12.110909. 41480161 PMC12754107

[25] Chłopek M , Lasota J , Singh O , Blakely AM , Glod J , Ylaya K , et al. DNA methylation profiling separates SDH-deficient GISTs from KIT-PDGFRA-driven GISTs and identifies predictive biomarkers for targeted therapy. Am J Surg Pathol. 2025; 49( 11): 1105– 13. doi:10.1097/PAS.0000000000002444. 40629847

[26] Nguyen THH , Vu GH , Nguyen TT , Nguyen TA , Tran VU , Vu LT , et al. Combination of hotspot mutations with methylation and fragmentomic profiles to enhance multi-cancer early detection. Cancer Med. 2025; 14: e70575. doi:10.1002/cam4.70575. 39748775 PMC11695824

[27] Takeuchi C , Yamashita S , Liu YY , Takeshima H , Sasaki A , Fukuda M , et al. Precancerous nature of intestinal *Metaplasia* with increased chance of conversion and accelerated DNA methylation. Gut. 2024; 73( 2): 255– 67. doi:10.1136/gutjnl-2023-329492. 37751933

[28] Capparelli R , Iannelli D . Epigenetics and *Helicobacter pylori*. Int J Mol Sci. 2022; 23( 3): 1759. doi:10.3390/ijms23031759. 35163679 PMC8836069

[29] Tahara S , Tahara T , Horiguchi N , Kato T , Shinkai Y , Yamashita H , et al. DNA methylation accumulation in gastric mucosa adjacent to cancer after *Helicobacter pylori* eradication. Int J Cancer. 2019; 144( 1): 80– 8. doi:10.1002/ijc.31667. 29978464

[30] Zheng SY , Zhu L , Wu LY , Liu HR , Ma XP , Li Q , et al. *Helicobacter pylori*-positive chronic atrophic gastritis and cellular senescence. Helicobacter. 2023;28:e12944. doi:10.1111/hel.12944. 36539375

[31] Muhammad JS , Manzoor S , Cui ZG , Khoder G . DNA methylation-mediated overexpression of CXCL1 in *Helicobacter pylori*-induced gastric cancer: *In silico*- and *in vitro*-based identification of a potential biomarker for carcinogenesis. Int J Mol Sci. 2023; 24( 1): 795. doi:10.3390/ijms24010795. 36614235 PMC9820856

[32] Baba Y , Ishimoto T , Kurashige J , Iwatsuki M , Sakamoto Y , Yoshida N , et al. Epigenetic field cancerization in gastrointestinal cancers. Cancer Lett. 2016; 375( 2): 360– 6. doi:10.1016/j.canlet.2016.03.009. 26971491

[33] Perri F , Cotugno R , Piepoli A , Merla A , Quitadamo M , Gentile A , et al. Aberrant DNA methylation in non-neoplastic gastric mucosa of *H. pylori* infected patients and effect of eradication. Am J Gastroenterol. 2007; 102( 7): 1361– 71. doi:10.1111/j.1572-0241.2007.01284.x. 17509026

[34] Shiotani A , Cen P , Graham DY . Eradication of gastric cancer is now both possible and practical. Semin Cancer Biol. 2013; 23( 6): 492– 501. doi:10.1016/j.semcancer.2013.07.004. 23876852

[35] Kim MJ , Kim HN , Jacobs JP , Yang HJ . Combined DNA methylation and gastric microbiome marker predicts *Helicobacter pylori*-negative gastric cancer. Gut Liver. 2024; 18( 4): 611– 20. doi:10.5009/gnl230348. 38509701 PMC11249944

[36] Okuno K , Joshi A , Watanabe S , Oba S , Yao K , Tanioka T , et al. Cell-free DNA methylation-based liquid biopsy assay to identify lymph node metastasis in T1 gastric cancer. UEG Journal. 2025; 13( 10): 2023– 33. doi:10.1002/ueg2.70132. 41105505 PMC12704565

[37] Savitri CMA , Rahmawati LD , Romadhon PZ , Waskito LA , Hidayat AA , Rezkitha YAA , et al. Epigenetic alterations in *Helicobacter pylori* infection leading to gastric carcinogenesis: A systematic review. J Res Med Sci. 2025; 30( 1): 50. doi:10.4103/jrms.jrms_705_22. 41122442 PMC12536862

[38] Han L , Shu X , Wang J . *Helicobacter pylori*-mediated oxidative stress and gastric diseases: A review. Front Microbiol. 2022; 13: 811258. doi:10.3389/fmicb.2022.811258. 35211104 PMC8860906

[39] Vahidi S , Mirzajani E , Norollahi SE , Aziminezhad M , Samadani AA . Performance of DNA Methylation on the Molecular Pathogenesis of *Helicobacter pylori* in Gastric Cancer; targeted therapy approach. J Pharmacopuncture. 2022; 25( 2): 88– 100. doi:10.3831/KPI.2022.25.2.88. 35837145 PMC9240405

[40] Ke B , Jin P , Wang XJ , Zhang RP , Liu N , Ma G . Pan-cancer landscape of ITGAV and its potential role in gastric cancer. Sci Rep. 2025; 15: 28934. doi:10.1038/s41598-025-14342-z. 40775431 PMC12332073

[41] Shahabi S , Kumaran V , Castillo J , Cong Z , Nandagopal G , Mullen DJ , et al. *LINC00261* is an epigenetically regulated tumor suppressor essential for activation of the DNA damage response. Cancer Res. 2019;79( 12): 3050– 62. doi:10.1158/0008-5472.CAN-18-2034. 30796052 PMC6571057

[42] Murray-Stewart T , Sierra JC , Piazuelo MB , Mera RM , Chaturvedi R , Bravo LE , et al. Epigenetic silencing of miR-124 prevents spermine oxidase regulation: Implications for *Helicobacter pylori*-induced gastric cancer. Oncogene. 2016; 35( 42): 5480– 8. doi:10.1038/onc.2016.91. 27041578 PMC5050049

[43] Zhang W , Cui N , Ye J , Yang B , Sun Y , Kuang H . Curcumin’s prevention of inflammation-driven early gastric cancer and its molecular mechanism. Chin Herb Med. 2022; 14( 2): 244– 53. doi:10.1016/j.chmed.2021.11.003. 36117672 PMC9476644

[44] Schneider BG , Peng DF , Camargo MC , Piazuelo MB , Sicinschi LA , Mera R , et al. Promoter DNA hypermethylation in gastric biopsies from subjects at high and low risk for gastric cancer. Int J Cancer. 2010; 127( 11): 2588– 97. doi:10.1002/ijc.25274. 20178103 PMC2916942

[45] Sobota RS , Kodaman N , Mera R , Piazuelo MB , Bravo LE , Pazos A , et al. Epigenetic and genetic variation in GATA5 is associated with gastric disease risk. Hum Genet. 2016; 135( 8): 895– 906. doi:10.1007/s00439-016-1687-1. 27225266 PMC4947561

[46] Schneider BG , Piazuelo MB , Sicinschi LA , Mera R , Peng DF , Roa JC , et al. Virulence of infecting *Helicobacter pylori* strains and intensity of mononuclear cell infiltration are associated with levels of DNA hypermethylation in gastric mucosae. Epigenetics. 2013; 8( 11): 1153– 61. doi:10.4161/epi.26072. 24128875 PMC3927747

[47] Holmes K , Egan B , Swan N , O’Morain C . Genetic mechanisms and aberrant gene expression during the development of gastric intestinal *Metaplasia* and adenocarcinoma. Curr Genom. 2007; 8( 6): 379– 97. doi:10.2174/138920207783406460. PMC2671722 19412438

[48] Sugai T , Sugimoto R , Habano W , Endoh M , Eizuka M , Tsuchida K , et al. Genetic differences stratified by PCR-based microsatellite analysis in gastric intramucosal neoplasia. Gastric Cancer. 2017; 20( 2): 286– 96. doi:10.1007/s10120-016-0616-2. 27236438

[49] Branham MT , Pellicer M , Campoy E , Palma M , Correa A , Roqué M . Epigenetic alterations in a gastric leiomyoma. Case Rep Gastrointest Med. 2014; 2014: 371638. doi:10.1155/2014/371638. 25544907 PMC4273588

[50] Do Nascimento Borges B , Burbano RM , Harada ML . Absence of CIP1/KIP1 hypermethylation in gastric cancer patients from Northern Brazil. In Vivo. 2010; 24( 4): 579– 82. 20668328

[51] Zhang T , Hu L , Zhang B , Tang X . Spasmolytic Polypeptide-Expressing Metaplasia (SPEM): The hidden linchpin in gastric carcinogenesis beyond the correa cascade. Int J Surg. 2026; 112(3):7951–8035. doi:10.1097/JS9.0000000000004273. 41347923

[52] Li F , Wang Y , Ping X , Yin JC , Wang F , Zhang X , et al. Molecular evolution of intestinal-type early gastric cancer according to *Correa* cascade. J Biomed Res. 2025; 39( 3): 270. doi:10.7555/JBR.38.20240118. PMC12239980 39314047

[53] Drebin HM , Walch H , Da Silva EM , Shimada S , Chatila W , Abate M , et al. Genomic signatures of gastric adenocarcinoma by tumor location-modified laurén classification: Highlighting the molecular heterogeneity of mixed-type tumors. Am J Surg Pathol. 2025; 49( 9): 948– 55. doi:10.1097/PAS.0000000000002432. 40512601

[54] Karlsson K , Przybilla MJ , Kotler E , Khan A , Xu H , Karagyozova K , et al. Deterministic evolution and stringent selection during preneoplasia. Nature. 2023; 618( 7964): 383– 93. doi:10.1038/s41586-023-06102-8. 37258665 PMC10247377

[55] Wong CC , Kang W , Xu J , Qian Y , Luk STY , Chen H , et al. Prostaglandin E_2_ induces DNA hypermethylation in gastric cancer *in vitro* and *in vivo*. Theranostics. 2019; 9( 21): 6256– 68. doi:10.7150/thno.35766. 31534549 PMC6735505

[56] Shin CM , Kim N , Jung Y , Park JH , Kang GH , Kim JS , et al. Role of *Helicobacter pylori* infection in aberrant DNA methylation along multistep gastric carcinogenesis. Cancer Sci. 2010; 101( 6): 1337– 46. doi:10.1111/j.1349-7006.2010.01535.x. 20345486 PMC11159191

[57] Liu C , Wu CH , Jia YB , Qiu JX , Li XY , Ling JH . Gastric organoids: A promising model for studying “inflammation-cancer” transition in atrophic gastritis. World J Clin Oncol. 2025; 16( 11): 110453. doi:10.5306/wjco.v16.i11.110453. 41355914 PMC12678949

[58] Yang J , Peng Y , Ding Y , Liu Y , Wang Y , Liu Y , et al. The clinicopathological and molecular characteristics of endocervical gastric-type adenocarcinoma and the use of Claudin18.2 as a potential therapeutic target. Mod Pathol. 2024; 37( 10): 100569. doi:10.1016/j.modpat.2024.100569. 39025403

[59] Landau DA , Clement K , Ziller MJ , Boyle P , Fan J , Gu H , et al. Locally disordered methylation forms the basis of intratumor methylome variation in chronic lymphocytic leukemia. Cancer Cell. 2014; 26( 6): 813– 25. doi:10.1016/j.ccell.2014.10.012. 25490447 PMC4302418

[60] Mazor T , Pankov A , Johnson BE , Hong C , Hamilton EG , Bell RJA , et al. DNA methylation and somatic mutations converge on the cell cycle and define similar evolutionary histories in brain tumors. Cancer Cell. 2015; 28( 3): 307– 17. doi:10.1016/j.ccell.2015.07.012. 26373278 PMC4573399

[61] Boyes J , Bird A . DNA methylation inhibits transcription indirectly via a methyl-CpG binding protein. Cell. 1991; 64( 6): 1123– 34. doi:10.1016/0092-8674(91)90267-3. 2004419

[62] Easwaran H , Johnstone SE , Van Neste L , Ohm J , Mosbruger T , Wang Q , et al. A DNA hypermethylation module for the stem/progenitor cell signature of cancer. Genome Res. 2012; 22( 5): 837– 49. doi:10.1101/gr.131169.111. 22391556 PMC3337430

[63] Kwon MK , Park G , Go D , Park D , Hannenhalli S , Choi SS . Transcription factors overcome the repressive impact of Polycomb-associated methylation in tumors. bioRxiv. 2025. doi:10.1101/2025.05.15.654174.

[64] Cedar H , Bergman Y . Linking DNA methylation and histone modification: Patterns and paradigms. Nat Rev Genet. 2009; 10( 5): 295– 304. doi:10.1038/nrg2540. 19308066

[65] De Marco K , Sanese P , Simone C , Grossi V . Histone and DNA methylation as epigenetic regulators of DNA damage repair in gastric cancer and emerging therapeutic opportunities. Cancers. 2023; 15( 20): 4976. doi:10.3390/cancers15204976. 37894343 PMC10605360

[66] Morales-Sanchez A , Fuentes-Panana EM . Epstein-Barr virus-associated gastric cancer and potential mechanisms of oncogenesis. Curr Cancer Drug Targets. 2017; 17( 6): 534– 54. doi:10.2174/1568009616666160926124923. 27677953

[67] Nishizawa T , Suzuki H . Gastric carcinogenesis and underlying molecular mechanisms: *Helicobacter pylori* and novel targeted therapy. BioMed Res Int. 2015; 2015: 794378. doi:10.1155/2015/794378. 25945346 PMC4405013

[68] Saliminejad K , Fard SS , Khorshid HRK , Yaghmaie M , Mahmoodzadeh H , Mousavi SA , et al. Methylation Analysis of P16, RASSF1A, RPRM, and RUNX3 in Circulating Cell-Free DNA for Detection of Gastric Cancer: A Validation Study. Avicenna J Med Biotechnol. 2020; 12( 2): 99– 106. 32431794 PMC7229449

[69] Kurklu B , Whitehead RH , Ong EK , Minamoto T , Fox JG , Mann JR , et al. Lineage-specific RUNX3 hypomethylation marks the preneoplastic immune component of gastric cancer. Oncogene. 2015; 34( 22): 2856– 66. doi:10.1038/onc.2014.233. 25088199 PMC4401645

[70] Graziano F , Humar B , Guilford P . The role of the E-cadherin gene (*CDH1*) in diffuse gastric cancer susceptibility: From the laboratory to clinical practice. Ann Oncol. 2003; 14( 12): 1705– 13. doi:10.1093/annonc/mdg486. 14630673

[71] Li Y , Yang Y , Lu Y , Herman JG , Brock MV , Zhao P , et al. Predictive value of CHFR and MLH1 methylation in human gastric cancer. Gastric Cancer. 2015; 18( 2): 280– 7. doi:10.1007/s10120-014-0370-2. 24748501 PMC4894312

[72] Spagnol LW , Polettini J , Silveira DA , Wegner GRM , Paiva DFF . P16 gene promoter methylation is associated with oncogenesis and progression of gastric carcinomas: A systematic review and meta-analysis. Crit Rev Oncol. 2022; 180: 103843. doi:10.1016/j.critrevonc.2022.103843. 36270449

[73] Lee Y . Change in gene expression profiles of secreted frizzled-related proteins (SFRPs) by sodium butyrate in gastric cancers: Induction of promoter demethylation and histone modification causing inhibition of Wnt signaling. Int J Oncol. 2012; 40( 5): 1533– 42. doi:10.3892/ijo.2012.1327. 22246241

[74] Kim W , Son H , Park J , Song S , Park C . Promoter methylation and down-regulation of DAPK is associated with gastric atrophy. Int J Mol Med. 2003; 12( 6): 827– 30. doi:10.3892/ijmm.12.6.827. 14612952

[75] Yu D , Cao T , Han YD , Huang FS . Relationships between *MGMT* promoter methylation and gastric cancer: A meta-analysis. OncoTargets Ther. 2016; 9: 6049– 57. doi:10.2147/OTT.S114052. PMC5063565 27785051

[76] Gao YJ , Xin Y , Zhang JJ , Zhou J . Mechanism and pathobiologic implications of CHFR promoter methylation in gastric carcinoma. World J Gastroenterol. 2008; 14( 32): 5000. doi:10.3748/wjg.14.5000. 18763281 PMC2742926

[77] Endoh M , Tamura G , Honda T , Homma N , Terashima M , Nishizuka S , et al. RASSF_2_, a potential tumour suppressor, is silenced by CpG island hypermethylation in gastric cancer. Br J Cancer. 2005; 93( 12): 1395– 9. doi:10.1038/sj.bjc.6602854. 16265349 PMC2361541

[78] Weber M , Hellmann I , Stadler MB , Ramos L , Pääbo S , Rebhan M , et al. Distribution, silencing potential and evolutionary impact of promoter DNA methylation in the human genome. Nat Genet. 2007; 39( 4): 457– 66. doi:10.1038/ng1990. 17334365

[79] Sproul D , Nestor C , Culley J , Dickson JH , Dixon JM , Harrison DJ , et al. Transcriptionally repressed genes become aberrantly methylated and distinguish tumors of different lineages in breast cancer. Proc Natl Acad Sci U S A. 2011; 108( 11): 4364– 9. doi:10.1073/pnas.1013224108. 21368160 PMC3060255

[80] Jones PA . Functions of DNA methylation: Islands, start sites, gene bodies and beyond. Nat Rev Genet. 2012; 13( 7): 484– 92. doi:10.1038/nrg3230. 22641018

[81] Liu MC , Oxnard GR , Klein EA , Swanton C , Seiden MV , Liu MC , et al. Sensitive and specific multi-cancer detection and localization using methylation signatures in cell-free DNA. Ann Oncol. 2020; 31( 6): 745– 59. doi:10.1016/j.annonc.2020.02.011. 33506766 PMC8274402

[82] Liu Y , Sethi NS , Hinoue T , Schneider BG , Cherniack AD , Sanchez-Vega F , et al. Comparative Molecular Analysis of Gastrointestinal Adenocarcinomas. Cancer Cell. 2018; 33( 4): 721– 35.e8. doi:10.1016/j.ccell.2018.03.010. 29622466 PMC5966039

[83] Yamada H , Takeshima H , Fujiki R , Yamashita S , Sekine S , Ando T , et al. ARID1A loss-of-function induces CpG island methylator phenotype. Cancer Lett. 2022; 532: 215587. doi:10.1016/j.canlet.2022.215587. 35131383

[84] Kim H , Kim YH , Kim SE , Kim NG , Noh SH , Kim H . Concerted promoter hypermethylation of *hMLH1*, *p16^INK4A^*, and *E-cadherin* in gastric carcinomas with microsatellite instability. J Pathol. 2003; 200( 1): 23– 31. doi:10.1002/path.1325. 12692837

[85] Nowak E , Bednarek I . Aspects of the epigenetic regulation of EMT related to cancer metastasis. Cells. 2021; 10( 12): 3435. doi:10.3390/cells10123435. 34943943 PMC8700111

[86] Wu BK , Mei SC , Brenner C . RFTS-deleted DNMT1 enhances tumorigenicity with focal hypermethylation and global hypomethylation. Cell Cycle. 2014; 13( 20): 3222– 31. doi:10.4161/15384101.2014.950886. 25485502 PMC4615144

[87] Toyota M , Ahuja N , Suzuki H , Itoh F , Ohe-Toyota M , Imai K , et al. Aberrant methylation in gastric cancer associated with the CpG island methylator phenotype. Cancer Res. 1999; 59(21):5438–42. 10554013

[88] Jung YC , Hong SJ , Kim YH , Kim SJ , Kang SJ , Choi SW , et al. Chromosomal losses are associated with hypomethylation of the gene-control regions in the stomach with a low number of active genes. J Korean Med Sci. 2008; 23( 6): 1068. doi:10.3346/jkms.2008.23.6.1068. 19119454 PMC2612760

[89] Rawla P , Barsouk A . Epidemiology of gastric cancer: Global trends, risk factors and prevention. Prz Gastroenterol. 2019; 14( 1): 26– 38. doi:10.5114/pg.2018.80001. 30944675 PMC6444111

[90] Chen X , Gole J , Gore A , He Q , Lu M , Min J , et al. Non-invasive early detection of cancer four years before conventional diagnosis using a blood test. Nat Commun. 2020; 11: 3475. doi:10.1038/s41467-020-17316-z. 32694610 PMC7374162

[91] Watanabe Y , Kim HS , Castoro RJ , Chung W , Estecio MRH , Kondo K , et al. Sensitive and specific detection of early gastric cancer with DNA methylation analysis of gastric washes. Gastroenterology. 2009; 136( 7): 2149– 58. doi:10.1053/j.gastro.2009.02.085. 19375421 PMC2722957

[92] Nie Y , Gao X , Cai X , Wu Z , Liang Q , Xu G , et al. Combining methylated SEPTIN9 and RNF180 plasma markers for diagnosis and early detection of gastric cancer. Cancer Commun. 2023; 43( 11): 1275– 9. doi:10.1002/cac2.12478. PMC10631480 37584087

[93] Warnecke PM , Stirzaker C , Melki JR , Millar DS , Paul CL , Clark SJ . Detection and measurement of PCR bias in quantitative methylation analysis of bisulphite-treated DNA. Nucleic Acids Res. 1997; 25( 21): 4422– 6. doi:10.1093/nar/25.21.4422. 9336479 PMC147052

[94] Ji L , Sasaki T , Sun X , Ma P , Lewis ZA , Schmitz RJ . Methylated DNA is over-represented in whole-genome bisulfite sequencing data. Front Genet. 2014; 5: 341. doi:10.3389/fgene.2014.00341. 25374580 PMC4204604

[95] de Abreu AR , Ibrahim J , Lemonidis V , Mateiu L , Van Camp G , Op de Beeck K . Comparison of current methods for genome-wide DNA methylation profiling. Epigenet Chromatin. 2025; 18( 1): 57. doi:10.1186/s13072-025-00616-3. PMC12376410 40855329

[96] Li LC , Dahiya R . MethPrimer: Designing primers for methylation PCRs. Bioinformatics. 2002; 18( 11): 1427– 31. doi:10.1093/bioinformatics/18.11.1427. 12424112

[97] Warnecke PM , Stirzaker C , Song J , Grunau C , Melki JR , Clark SJ . Identification and resolution of artifacts in bisulfite sequencing. Methods. 2002; 27( 2): 101– 7. doi:10.1016/S1046-2023(02)00060-9. 12095266

[98] Seo SY , Youn SH , Bae JH , Lee SH , Lee SY . Detection and characterization of methylated circulating tumor DNA in gastric cancer. Int J Mol Sci. 2024; 25( 13): 7377. doi:10.3390/ijms25137377. 39000483 PMC11242052

[99] Nagano S , Kurokawa Y , Hagi T , Yoshioka R , Takahashi T , Saito T , et al. Extensive methylation analysis of circulating tumor DNA in plasma of patients with gastric cancer. Sci Rep. 2024; 14: 30739. doi:10.1038/s41598-024-79252-y. 39730450 PMC11680901

[100] Morris MJ , Hesson LB , Youngson NA . Non-CpG methylation biases bisulphite PCR towards low or unmethylated mitochondrial DNA: Recommendations for the field. Environ Epigenet. 2020; 6: dvaa001. doi:10.1093/eep/dvaa001. 32154030 PMC7055202

[101] Wen X , Pu H , Liu Q , Guo Z , Luo D . Circulating tumor DNA—A novel biomarker of tumor progression and its favorable detection techniques. Cancers. 2022; 14( 24): 6025. doi:10.3390/cancers14246025. 36551512 PMC9775401

[102] Suzuki T , Yoshida K , Wada Y , Hamai Y , Sentani K , Oue N , et al. Melanoma-associated antigen-A1 expression predicts resistance to docetaxel and paclitaxel in advanced and recurrent gastric cancer. Oncol Rep. 2007; 18( 2): 329– 36. doi:10.3892/or.18.2.329. 17611652

[103] Sawaki K , Kanda M , Kodera Y . Review of recent efforts to discover biomarkers for early detection, monitoring, prognosis, and prediction of treatment responses of patients with gastric cancer. Expert Rev Gastroenterol Hepatol. 2018; 12( 7): 657– 70. doi:10.1080/17474124.2018.1489233. 29902383

[104] Guo Y , Yu H , Li J , Liu K , Han M , Tang Y , et al. DNA-methylation eraser TET2 activates WTIP expression to suppress an AKT-dependent chemoresistance of gastric cancer. Neoplasia. 2025; 65: 101166. doi:10.1016/j.neo.2025.101166. 40279682 PMC12434994

[105] Lee JY , Nam M , Son HY , Hyun K , Jang SY , Kim JW , et al. Polyunsaturated fatty acid biosynthesis pathway determines ferroptosis sensitivity in gastric cancer. Proc Natl Acad Sci U S A. 2020; 117( 51): 32433– 42. doi:10.1073/pnas.2006828117. 33288688 PMC7768719

[106] Bae HJ , Kang SK , Kwon WS , Jeong I , Park S , Kim TS , et al. p16 methylation is a potential predictive marker for abemaciclib sensitivity in gastric cancer. Biochem Pharmacol. 2021; 183: 114320. doi:10.1016/j.bcp.2020.114320. 33161023

[107] Preston-Alp S , Caruso LB , Su C , Keith K , Soldan SS , Maestri D , et al. Decitabine disrupts EBV genomic epiallele DNA methylation patterns around CTCF binding sites to increase chromatin accessibility and lytic transcription in gastric cancer. mBio. 2023; 14( 5): e00396– 23. doi:10.1128/mbio.00396-23. 37606370 PMC10653948

[108] Servetas SL , Bridge DR , Merrell DS . Molecular mechanisms of gastric cancer initiation and progression by *Helicobacter pylori*. Curr Opin Infect Dis. 2016; 29( 3): 304– 10. doi:10.1097/QCO.0000000000000248. 26779778 PMC5144489

[109] Cao CQ , Chang L , Wu Q . Circulating methylated Septin 9 and ring finger protein 180 for noninvasive diagnosis of early gastric cancer. Transl Cancer Res. 2020; 9( 11): 7012– 21. doi:10.21037/tcr-20-1330. 35117307 PMC8799148

[110] Ko K , Kananazawa Y , Yamada T , Kakinuma D , Matsuno K , Ando F , et al. Methylation status and long-fragment cell-free DNA are prognostic biomarkers for gastric cancer. Cancer Med. 2021; 10( 6): 2003– 12. doi:10.1002/cam4.3755. 33641249 PMC7957189

[111] Chen ZH , Yan SM , Chen XX , Zhang Q , Liu SX , Liu Y , et al. The genomic architecture of EBV and infected gastric tissue from precursor lesions to carcinoma. Genome Med. 2021; 13: 146. doi:10.1186/s13073-021-00963-2. 34493320 PMC8422682

[112] Wang H , Wang X , Xu L , Zhang J , Cao H . High expression levels of pyrimidine metabolic rate–limiting enzymes are adverse prognostic factors in lung adenocarcinoma: A study based on The Cancer Genome Atlas and Gene Expression Omnibus datasets. Purinergic Signal. 2020; 16( 3): 347– 66. doi:10.1007/s11302-020-09711-4. 32638267 PMC7524999

[113] Corallo S , Lasagna A , Filippi B , Alaimo D , Tortorella A , Serra F , et al. Unlocking the potential: Epstein-Barr virus (EBV) in gastric cancer and future treatment prospects, a literature review. Pathogens. 2024; 13( 9): 728. doi:10.3390/pathogens13090728. 39338919 PMC11435077

[114] Agrawal K , Das V , Vyas P , Hajdúch M . Nucleosidic DNA demethylating epigenetic drugs–A comprehensive review from discovery to clinic. Pharmacol Ther. 2018; 188: 45– 79. doi:10.1016/j.pharmthera.2018.02.006. 29454856

[115] Wenger V , Garcia-Manero G , Zeiser R , Lübbert M . DNAmethyltransferase inhibitors in hematological malignancies and solid tumors. Int J Cancer. 2026; 158( 2): 433– 61. doi:10.1002/ijc.70197. 41204420 PMC12628039

[116] Li Z , Lei H , Luo M , Wang Y , Dong L , Ma Y , et al. DNA methylation downregulated mir-10b acts as a tumor suppressor in gastric cancer. Gastric Cancer. 2015; 18( 1): 43– 54. doi:10.1007/s10120-014-0340-8. 24481854

[117] Ganai SA , Sheikh FA , Baba ZA , Mir MA , Mantoo MA , Yatoo MA . Anticancer activity of the plant flavonoid luteolin against preclinical models of various cancers and insights on different signalling mechanisms modulated. Phytother Res. 2021; 35( 7): 3509– 32. doi:10.1002/ptr.7044. 33580629

[118] Niwa T , Toyoda T , Tsukamoto T , Mori A , Tatematsu M , Ushijima T . Prevention of *Helicobacter pylori*–induced gastric cancers in gerbils by a DNA demethylating agent. Cancer Prev Res. 2013; 6( 4): 263– 70. doi:10.1158/1940-6207.CAPR-12-0369. 23559452

[119] Biagioni A , Staderini F , Peri S , Versienti G , Schiavone N , Cianchi F , et al. 5-fluorouracil conversion pathway mutations in gastric cancer. Biology. 2020; 9( 9): 265. doi:10.3390/biology9090265. 32887417 PMC7563957

[120] Zhu L , Zhu Y , Li F , Meng Y , Wang H , Xu W , et al. RNautophagic regulation of DNMT3a-dependent DNA methylation by Linc00942 enhances chemoresistance in gastric cancer. Clin Transl Med. 2023; 13( 7): e1337. doi:10.1002/ctm2.1337. 37477089 PMC10359971

[121] Yan W , Wu K , Herman JG , Brock MV , Zhou Y , Lu Y , et al. Epigenetic silencing of DACH1 induces the invasion and metastasis of gastric cancer by activating TGF-β signalling. J Cell Mol Med. 2014; 18( 12): 2499– 511. doi:10.1111/jcmm.12325. 24912879 PMC4302654

[122] Chiappinelli KB , Strissel PL , Desrichard A , Li H , Henke C , Akman B , et al. Inhibiting DNA methylation causes an interferon response in cancer via dsRNA including endogenous retroviruses. Cell. 2015; 162( 5): 974– 86. doi:10.1016/j.cell.2015.07.011. 26317466 PMC4556003

[123] Fattahi S , Golpour M , Amjadi-Moheb F , Sharifi-Pasandi M , Khodadadi P , Pilehchian-Langroudi M , et al. DNA methyltransferases and gastric cancer: Insight into targeted therapy. Epigenomics. 2018; 10( 11): 1477– 97. doi:10.2217/epi-2018-0096. 30325215

[124] Li X , Li Y , Dong L , Chang Y , Zhang X , Wang C , et al. Decitabine priming increases anti–PD-1 antitumor efficacy by promoting CD8^+^ progenitor exhausted T cell expansion in tumor models. J Clin Investig. 2023; 133( 7): e165673. doi:10.1172/JCI165673. 36853831 PMC10065084

[125] Zhou Q , Xie Q , Liu Q , Wang H , Zhang Z , Yu Z , et al. DNA methylation inhibitors adverse reaction characteristic analysis: A descriptive analysis from WHO-VigiAccess. Front Pharmacol. 2024; 15: 1470148. doi:10.3389/fphar.2024.1470148. 39415836 PMC11479969

[126] Wolff F , Leisch M , Greil R , Risch A , Pleyer L . The double-edged sword of (re)expression of genes by hypomethylating agents: From viral mimicry to exploitation as priming agents for targeted immune checkpoint modulation. Cell Commun Signal. 2017; 15( 1): 13. doi:10.1186/s12964-017-0168-z. 28359286 PMC5374693

[127] Maeda O , Matsuoka A , Furukawa K , Miyahara R , Hirooka Y , Ando Y . Alterations in gene expression and DNA methylation profiles in gastric cancer cells obtained from ascitic fluids collected before and after chemotherapy. Mol Clin Onc. 2019; 11( 1): 91– 8. doi:10.3892/mco.2019.1858. PMC6543769 31289684

[128] Kodach LL , Jacobs RJ , Voorneveld PW , Wildenberg ME , Verspaget HW , van Wezel T , et al. Statins augment the chemosensitivity of colorectal cancer cells inducing epigenetic reprogramming and reducing colorectal cancer cell ‘stemness’ via the bone morphogenetic protein pathway. Gut. 2011; 60( 11): 1544– 53. doi:10.1136/gut.2011.237495. 21551187

[129] Nakajima T , Yamashita S , Maekita T , Niwa T , Nakazawa K , Ushijima T . The presence of a methylation fingerprint of *Helicobacter pylori* infection in human gastric mucosae. Int J Cancer. 2009; 124( 4): 905– 10. doi:10.1002/ijc.24018. 19035455

[130] Lee TB , Park JH , Min YD , Kim KJ , Choi CH . Epigenetic mechanisms involved in differential MDR1mRNA expression between gastric and colon cancer cell lines and rationales for clinical chemotherapy. BMC Gastroenterol. 2008; 8( 1): 33. doi:10.1186/1471-230X-8-33. 18673531 PMC2529328

